# ALOX5 regulates vascular smooth muscle cells pyroptosis to affect abdominal aortic aneurysm formation

**DOI:** 10.1038/s41598-025-14268-6

**Published:** 2025-08-09

**Authors:** Luobo Wang, Bing Wang

**Affiliations:** 1 Department of Vascular Surgery, The Fifth Affiliated Hospital of Zhengzhou University, Zhengzhou, 450052 China; 2 Department of Vascular Surgery, Jining NO.1 People’s Hospital, Jining, 272000 China

**Keywords:** Abdominal aortic aneurysm, Pyroptosis, ALOX5, NF-κB, Cell biology, Developmental biology, Biomarkers, Diseases

## Abstract

ALOX5 is a member of the lipoxygenase (LOX) family. It is ubiquitous in all tissues and cells of the human body and participates in cell metabolism, and is closely related to human inflammation and occurrence and development of various diseases. ALOX5 was reported to have a certain impact on occurrence and development of nervous system, respiratory system and cardiovascular system diseases. However, there are few reports on the effect of ALOX5 on abdominal aortic aneurysm (AAA). This study aims to clarify the relevant influence of ALOX5 on AAA occurrence or development, and explore the mechanism through which ALOX5 plays a role in AAA formation. The AAA mouse model was constructed by subcutaneous implantation of a permeable micropump and injection of angiotensin II. After different treatment, the morphology of abdominal artery was observed and photographed. The mean diameter of abdominal aorta and tumor formation rate were recorded in each group. The thickness and shape of blood vessel wall were observed by HE staining. Masson staining was used to observe distal vascular wall precipitation and vascular wall fibrosis. In the cell experiment, the level of related indexes was detected after adding angiotensin II to MA-VSMCs. The levels of inflammatory factors, ROS level, MDA level, SOD activity and LDH release were detected by ELISA. Related proteins were detected by Western blotting. ALOX5 mRNA level was assessed by RT-qPCR. Integrity of MA-VSMCs cell membrane was detected by Hoechst 33342/PI double staining kit. Ang-II induced ApoE^−/−^ mice to establish AAA model successfully. ALOX5 expression was increased in the ApoE^−/−^ mouse AAA model. Addition of ALOX5 inhibitor reduced the occurrence and severity of AAA as well as inflammatory, oxidative stress and pyroptotic protein levels in ApoE^−/−^ mice. Down-regulation of ALOX5 inhibited LDH release, oxidative stress, inflammation and pyroptosis of MA-VASMCs induced by Ang II. After ALOX5 inhibition, the NF-κB pathway was inhibited. ALOX5 overexpression promoted the release of LDH, PI-positive cells, pyroptosis related protein expression, oxidative stress and inflammatory cytokine release induced by Ang II in MA-VSMCs, while they were reversed by BAY11-7082 (BAY, NF-κB inhibitors). This study confirmed that ALOX5 involved in AAA development. Adding ALOX5 inhibitors to animal modeling could delay AAA development. Silencing or overexpressing ALOX5 in MA-VSMCs induced by Ang II correspondingly decreased or increased inflammatory cytokines and pyroptosis. The addition of NF-κB pathway inhibitor BAY inhibited the increase of inflammatory factors and pyroptosis caused by ALOX5 overexpression. These results indicated that ALOX5 promoted pyroptosis through NF-κB pathway, and then promoted AAA development.

**Supplementary Information:**

The online version contains supplementary material available at 10.1038/s41598-025-14268-6.

## Introduction

Abdominal aortic aneurysm (AAA) is a degenerative disease that presents as persistent dilation of the wall of the main artery, which is called AAA when the diameter of the blood vessels is more than 50% of the normal diameter^1^. AAA is a cardiovascular disease characterized by irreversible and progressive arterial dilation, which ultimately leads to aortic rupture and seriously affects the patient’s life safety^2^. There are currently no effective drugs to treat aneurysms, and surgery is the main way to save a patient’s life^3^. Open surgery is the traditional treatment of ruptured AAA, which is characterized by high trauma, high risk and low patient tolerance^4,5^. Intracavitary surgery has less trauma and quick recovery, but postoperative stent deformation, displacement, infection, internal leakage and other complications seriously affect the therapeutic effect^6^. The development of AAA is a complex process, and the main pathological changes include extracellular matrix (ECM) degradation, inflammation, vascular smooth muscle cells (VSMCs) apoptosis, oxidative stress and so on^2,7,8^. However, exact molecular mechanisms of AAA progression remain unclear. Thus, it is urgent to identify etiological mechanism of AAA occurrence and progression in order to provide new targets for AAA diagnosis and treatment.

AAA is characterized by inflammatory infiltration and oxidation-reduction system disorder^9^. Overexpression of inflammatory factors leads to local inflammatory cell infiltration during the onset, and the infiltrated inflammatory cells act on the oxidative system, causing oxidative stress and promoting the initiation and formation of AAA^10^. There are many kinds of immune cells involved in inflammation, such as lymphocytes, macrophages and mast cells^11^. Helper T lymphocyte 2 in lymphocytes can secrete IL-4, IL-10, IL-5, etc., thereby reducing the toxic effect of macrophages and the release of pro-inflammatory mediators^11–13^. Macrophages are the most common white blood cells in the aortic outer membrane, secreting transforming growth factor β and inflammatory factors such as IL-­1β, IL-6 and IL-8, which directly lead to the degradation of the extracellular matrix and promote the formation of AAA^11–13^. Oxidative stress can produce excessive reactive oxygen species (ROS), ROS can promote the secretion of inflammatory factors by inflammatory cells to induce the apoptosis of VSMCs, and activate matrix metalloproteinases to promote the degradation of extracellular matrix in the aortic wall and the activation of collagenase to cause the destruction of elastic fibers, thus weakening the elasticity of the vascular wall and eventually developing into aneurysms^14^. As one of the end products of lipid oxidation, malondialdehyde (MDA) can be used as an index of oxidation level in AAA^15^. Superoxide dismutase (SOD) can effectively eliminate superoxide anion radicals and plays an important role in oxidation and antioxidant processes of AAA^16^. Inflammation and oxidative stress are the pathological basis for the formation of aneurysms. Therefore, reducing or inhibiting chronic inflammatory reactions and minimizing oxidative stress damage are of great significance in exploring the pathogenesis and treatment of AAA.

Pyroptosis is a kind of inflammatory programmed cell death discovered in recent years, accompanied by the release of a variety of pro-inflammatory cytokines, which is closely related to inflammation^17^. Pyroptosis mainly manifested by the gradual expansion of cell volume after the formation of cell membrane cracks, which eventually leads to the cell membrane rupture and contents release, thus inducing the body to produce a strong inflammatory response^18^. There are mainly four pathways that have been identified to mediate pyroptosis, including the classical pathway mediated by Caspase-1, the non-classical pathway mediated by Caspase-4/5/11, the apoptosis-related caspase-mediated pathway and the granzyme (GZM) based pathway^19–21^. Pyroptosis relates to progression of metabolic diseases, tumors, immune-related diseases, cardiovascular diseases, neuro-inflammatory reactions and many other diseases^22^. Pyroptosis plays vital role in AAA progression^23,24^. VSMCs are an important component of the vascular wall, and their proliferation inhibition, apoptosis promotion and ECM degradation are key factors that promote the development of AAA^25,26^. As one of the modes of SMCs death, pyroptosis is of great significance not only for cardiovascular diseases, but also for the occurrence and development of multi-organ diseases^27–29^. At present, it is unclear whether pyroptosis is beneficial for the treatment of AAA, and more studies are needed to explore VSMCs pyroptosis’ role in AAA, which may provide direction for the treatment of AAA.

Arachidonic acid 5-lipoxygenase (ALOX5) is an iron-containing non-heme dioxygenase that catalyzes the peroxidation of polyunsaturated fatty acids such as arachidonic acid and plays vital role in inflammatory responses, cell death, and tumor occurrence and development^30^. ALOX5 has been implicated in many diseases development, including cardiovascular disease, allergic rhinitis, and tumors^31^. As the research on ALOX5 continues to deepen, its role in diseases progression will receive more and more attention. In recent years, ALOX5-mediated inflammation regulation mechanism and physiological pathogenesis of atherosclerosis have been paid more attention^32^. ALOX5 may become a promising therapeutic target for human cardiovascular diseases treatment^33^. ALOX5 expression was increased in human AAA tissues^34^. In addition, ALOX5 inhibition reduce the incidence of AAA and slow tumor growth after aneurysm formation in animal models^35^. This indicates that ALOX5 is closely related to AAA and has potential diagnostic value for AAA progression, but the specific mechanism of action is not completely clear. Therefore, this study constructed a mouse AAA model by subcutaneously implanting angiotensin II (Ang II) into ApoE^−/−^ mice and induced VSMCs pyroptosis by Ang II to explore how ALOX5 effect AAA progression, providing new targets for the treatment and drug development of AAA.

## Materials and methods

### Ethics statement

All animal work in the study followed ARRIVE guidelines. All experiments conducted in this study were in strict accordance with relevant guidelines and regulations. All animal work in the study followed ARRIVE guidelines and approved by the animal ethics of Beijing Xinuoyin Biotechnology Co., Ltd (XNY-2024-YX-033).

### Construction of mouse AAA model

SPF C57BL/6 ApoE^−/−^ (ApoE knock out, ApoE KO) male mice aged 10 to 12 weeks were provided by the Beijing Xinuoyin Biotechnology Co., Ltd [SYXK (Beijing) 2022-0024]. According to the latest animal experiment management measures of Beijing Xinuoyin Biotechnology Co., Ltd, experimental animals were raised in the SPF animal room of our laboratory for one week and then enrolled for corresponding operations.

The experimental mice were numbered and randomly divided into sham operation group (sham), model group (AAA), model and dimethyl sulfoxide (DMSO, solvent of ALOX5 inhibitor) group (AAA + DMSO) and ALOX5 inhibitor AZD4407 intervention group (AAA + inhibitor). Trace slow-release pump (1000 ng/kg/min) of Ang II was implanted by interscapular subcutaneous surgery in mice of AAA, AAA + DMSO and AAA + inhibitor group, while the sham group were replaced with equal volume of normal saline. The AAA + inhibitor group was treated with AZD4407 (30 mg/kg/day) every day after surgery, while the AAA + DMSO group were replaced with equal volume of DMSO. All mice were fed with free drinking water and standard diet. After feeding in groups for 28 days, the mice were anesthetized by isoflurane and killed. The morphology of abdominal artery was observed and photographed. The average diameter and tumor formation rate of abdominal aorta were recorded in each group. According to the needs of the experiment, the measurement was completed, and the arteries of the mice were either implanted with 4% paraformaldehyde, embedded with dehydrated paraffin, or stored in a frozen liquid nitrogen tank.

### Vascular ultrasound

Mice were anesthetized with 1% isoflurane and shaved from the chest to the abdomen. Ultrasound scanning is performed using the Mindray ultrasound system. The ultrasonic probe scanned the mouse aorta vertically in the abdomen of the mouse, adjusted the direction so that the ultrasonic image of the mouse’s abdominal aorta was located in the center of the display screen, collected the representative image at the maximum diameter of the suprarenal abdominal aorta, and measured the recorded data. After 28 days of modeling, the maximum diameter of the abdominal aorta was detected.

### Hematoxylin-eosin (HE) staining

The paraffin sections were baked in the oven for 30 min at 55℃. After the wax was soaked and dewaxed with xylene, the sections were rehydrated with gradient ethanol. The water on the sections was drained and then soaked in the hematoxylin dye solution for 10 min. After that, the sections were immersed in 1% dilute hydrochloric acid, until the tissue changed from purple to pink, cleaned with distilled water, then soaked in eosin dye for 1 min, rinsed with distilled water, dehydrated with gradient ethanol, soaked in xylene, and finally sealed with neutral resin. The sealed sections could be stored in a ventilator and dried for long-term use. The lumen and artery wall of the abdominal aorta were examined under microscope (SZ61, Olympus, Japan).

### Masson staining

The process of Masson staining baking sheet and dewaxing hydration is the same as HE staining. After drying the water on the glass slide, Masson’s complex staining solution was added for 5 min of staining. After rinsing with distilled water, phosphomolybdic acid was added for 5 min of staining. After wiping off the surface moisture of the slices, aniline blue was directly was added for 5 min of staining. After a slight rinse with distilled water, differentiation solution was added to differentiate with 30–60 s. Then, glass slide was rinsed with distilled water and added another drop of differentiation solution to differentiate with 30–60 s. The dehydration and sealing steps were the same as HE staining. The damage of elastic fibers in abdominal aorta was observed under microscope.

### Cell culture and treatment

Mouse aortic VSMCs (MA-VSMCs) purchased from Procell (Wuhan, China). MA-VSMCs were cultured in DMEM/F12 medium containing 10% FBS, 1% penicillin-streptomycin. The cells were incubated in a constant temperature incubator at 37℃ with 5% CO_2_. 5–10 passages of MA-VSMCs in good condition were selected for the experiment.

MA-VSMCs was seeded and cultured in 6-well cell culture plates. When cell density reached 70%, cells were treated with starvation without FBS medium for 24 h and then added to Ang II (1 µmol/L) (Ang group), respectively^36^. After culture for 0, 6, 12, 24, 48 h, cells were collected to carry out relevant indicator detection.

si-NC and si-ALOX5, pc-NC and pc-ALOX5 were produced by GenePharma (Shanghai, China). MA-VSMCs were divided into Ang group, Ang + si-NC group, Ang + pc-NC, Ang + pc-ALOX5 group and Ang + si-ALOX5 group. All groups were added with 1 µmol/L Ang II for 48 h, and Ang + si-NC group, Ang + si-ALOX5 group, Ang + si-NC group, and Ang + pc-ALOX5 group were transfected with Lipofectamine™ 3000 reagent for si-NC, si-ALOX5, pc-NC and pc-ALOX5, respectively. Cells were collected after 48 h transfection for follow-up experiments.

### RT-qPCR

The tissues and cells were collected after treatment, and the total RNA in tissues (fully ground by homogenizer) and cells were extracted by Trizol method. RNA concentration was detected by ultraviolet spectrophotometer. According to Primescript™ RT reagent kit instruction, sample of RNA was converted into cDNA. RT-qPCR was performed according to instruction of SYBR^®^ Premix Ex TaqTM II kit. mRNA expression level in tissues and cells of each group was calculated by 2^−ΔΔCt^ with GAPDH as internal parameter.

### Western blot

The frozen tissue and the treated cells were taken. Lysate was added to extract the total protein in tissue (fully ground by homogenizer) and cells. Protein concentration was detected by BCA kit. The sample to be tested was mixed with sample buffer and denatured in 100℃ water for 5 min. Then the sample was added to the prepared SDS-PAGE gel sample hole, each hole was 25 µL. The adjustment voltage was 60 V during glue concentration, and the separation voltage was 120 V. After the gel was removed, the bands were transferred on PVDF membrane at 4℃ for 1.5 h. PVDF membrane was sealed with 5% skim milk powder for 2 h, and the primary antibody was added at 4℃ overnight. After washing, secondary antibodies were added and incubated for 2 h at 37℃. With the addition of ECL development, an automatic gel imaging system was used to collect images with β-actin as internal reference to analyze protein levels.

### LDH release detection

Cells were inoculated into 96-well plates (2 × 10^5^ cells/well). When the cells were 70–80% confluent, they were replaced with serum-free medium for culture. After corresponding treatment or transfection, the cell culture plates were centrifuged with perforated plates. The supernatant of each hole was 120 µL and added to the corresponding hole of the new 96-well plate. LDH detection reagent (60 µL/well) was added, mixed several times, and incubated at 25℃ for 30 min away from light. LDH release in supernatant was detected using LDH detection kit.

### Hoechst/PI staining

MA-VSMCs was planted in 6-well plates. After corresponding treatment or transfection, cells were rinsed with PBS solution for 3 times. 5 µL Hoechst 33342 working solution and 5 µL PI working solution were added respectively. After gently mixing, MA-VSMCs was incubated for 30 min at 4℃ in the dark. Cells were rinsed with PBS solution twice, and the PI-positive cells was observed under fluorescence microscope.

### Detection of inflammatory factors

Before mice were killed, 2 ml blood was collected from the tail vein and centrifuged at 4℃ with 3000 r/min for 5 min, the upper serum was taken. Cells in good condition were inoculated into 6-well plates with cell density of about 4 × 10^5^ cells/mL, 2 mL was added to each well, and cell culture supernatant was collected for detection after culture. Contents of IL-1β, IL-6, IL-10 and IL-18 were detected with the kit, and the operation process was carried out with the instructions of the kit.

### Oxidative stress detection

Samples were obtained by the previous method treatment and ROS detection kit (DCFH-DA probe method) was used to detect ROS levels. Moreover, the oxidative stress level was measured MDA concentration and SOD activity. MDA was detected by thiobarbituric acid (TBA) with a maximum absorption peak of 532 nm wavelength. Nitro tetrazolium blue (NBT) was used to detect SOD activity with a maximum absorption peak of 412 nm.

### Statistical analysis

All data were processed by GraphPad Prism 8.0 software, and data processing was expressed as mean ± standard deviation (mean ± SD). T-test was used for comparison between two groups, and ANOVA was used for comparison between multiple groups, and *P <* 0.05 was considered statistically significant.

## Results

### ALOX5 inhibition alleviated AAA occurrence in mice

**Fig. 1:**
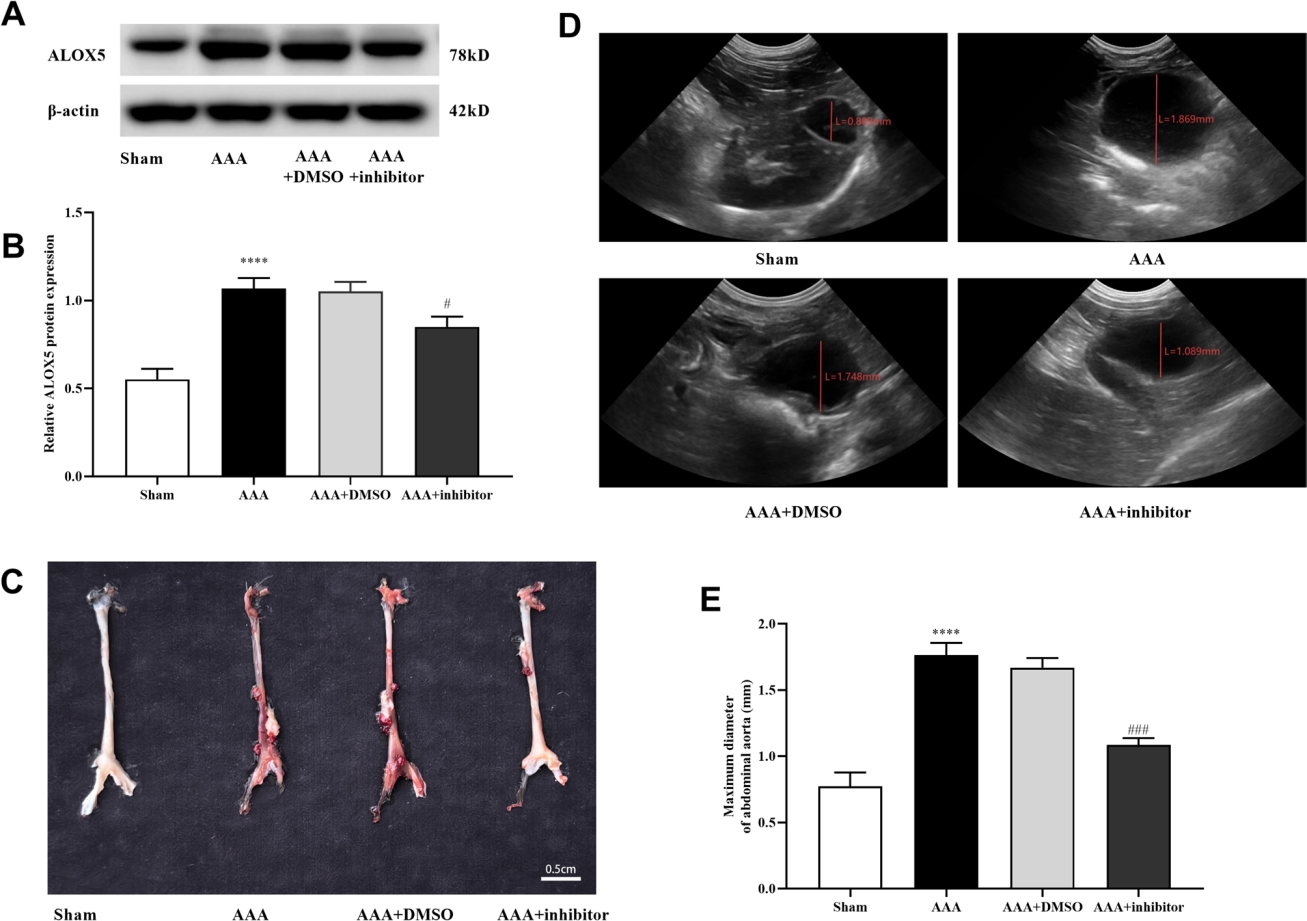
ALOX5 inhibition alleviated AAA occurrence in mice. (**A**, **B**) Expression of ALOX5 protein in abdominal aorta specimens of mice in each group. (**C**) Representative anatomical images of abdominal aorta in each group of mice. (**D**) Ultrasound images of abdominal aorta of each group after 28 days of treatment. (**E**) Comparison of the maximum diameter of abdominal aorta in each group. Ang II: 1000 ng/kg/min. ALOX5 inhibitor: 30 mg/kg/day. ****P < 0.0001, compared with sham group. #*P <* 0.05, ###*P <* 0.001, compared with AAA + DMSO group.

After 4 weeks, ALOX5 expression in AAA tissues was detected by Western blot. The results showed that ALOX5 expression in Ang II-induced AAA tissues was increased versus sham group, suggesting that ALOX5 was highly expressed in AAA tissues of Ang II-induced mice (Fig. 1A and B). After 28 days treatment, no aneurysms were formed in the sham group, but aneurysms mainly occurred in the superior renal area of the abdominal aorta and were characterized by swelling and transmural thrombosis in the other groups (Fig. 1C). Diameter of abdominal aorta in AAA group was increased versus sham group (Fig. 1D and E). However, diameter of abdominal aorta was reduced after inhibitors addition versus AAA + DMSO group (Fig. 1D and E). The above results indicated that inhibiting ALOX5 might limit the progressive dilation of the abdominal aortic lumen, thereby inhibiting AAA progression.

### ALOX5 inhibition improved pathological changes of AAA in mice

**Fig. 2:**
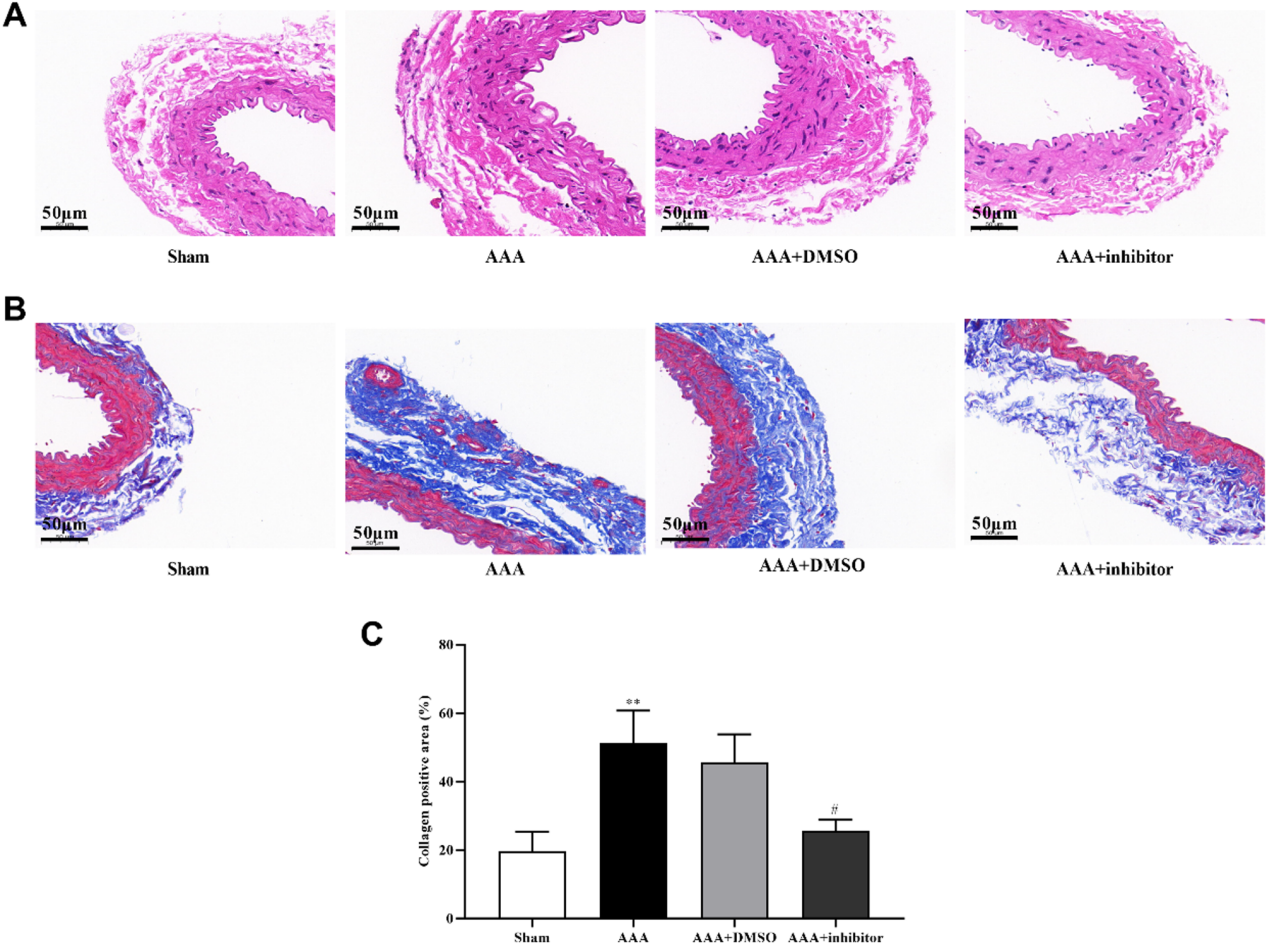
ALOX5 inhibition improved pathological changes of AAA in mice. (**A**) HE staining representation of abdominal aorta specimens of mice in each group. (**B**) Masson staining representation of abdominal aorta specimens in each group of mice. (**C**) The quantitative analysis of fibrotic areas of Masson’s staining. Ang II: 1000 ng/kg/min. ALOX5 inhibitor: 30 mg/kg/day. **P < 0.01, compared with sham group. #*P <* 0.05, compared with AAA + DMSO group.

To further clarify the success of the AAA model and the pathological changes in each experimental group, we performed HE and Masson staining on paraffin embedded sections of tumor specimens from each group. Structure of the vascular wall in AAA group and AAA + DSMO group was disordered and inflammatory cell infiltration was present versus sham group, while AAA + inhibitor showed relatively complete structure and reduction in inflammatory cell infiltration (Fig. 2A). Masson staining results showed that compared with sham group, a large amount of collagen fibers were deposited in AAA group and AAA + DSMO group, while less collagen fibers were deposited in AAA + inhibitor group (Fig. 2B and C). This suggested that ALOX5 inhibition improved the histopathological damage of the wall of abdominal aortic aneurysm in mice.

### ALOX5 inhibition decreased inflammatory factors and oxidative stress levels of AAA in mice

**Fig. 3:**
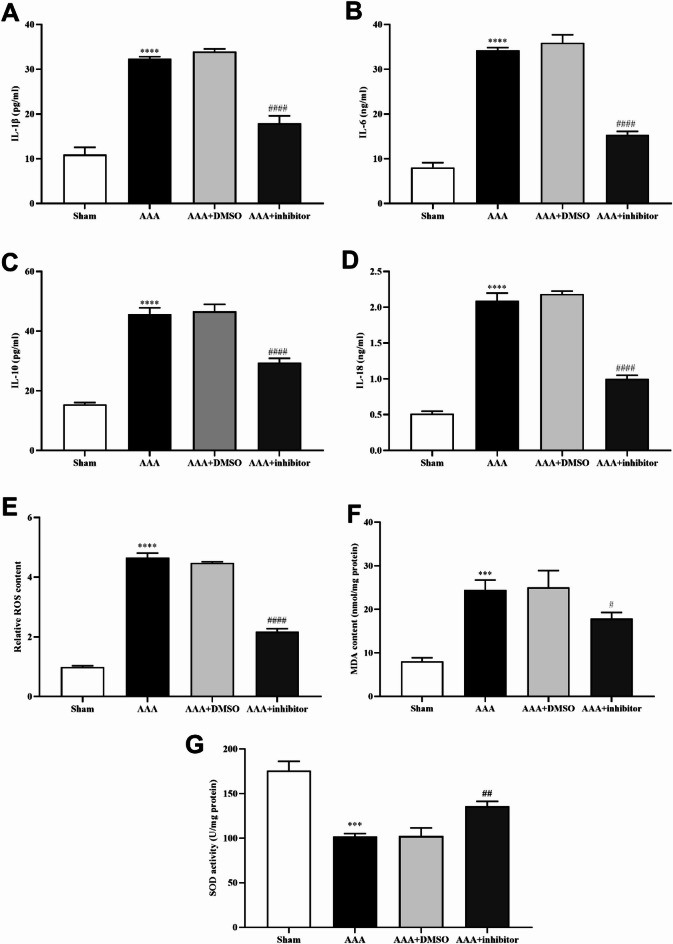
ALOX5 inhibition decreased inflammatory factors and oxidative stress levels of AAA in mice. (**A**, **B**, **C**, **D**) Levels of IL-1β, IL-6, IL-10 and IL-18 in AAA tissue in each group. (**E**) ROS levels in AAA tissues in each group. (**F**) MDA levels in AAA tissues in each group. (**G**) SOD activity in AAA tissues in each group. Ang II: 1000 ng/kg/min. ALOX5 inhibitor: 30 mg/kg/day. ***P < 0.001, ****P < 0.0001, compared with sham group. #*P <* 0.05, ##*P <* 0.01, ###*#P <* 0.0001, compared with AAA + DMSO group.

Inflammatory cell infiltration and oxidative stress play vital role in AAA occurrence and development. Thus, inflammatory factors level and oxidative stress in each group of mice were detected. The IL-1β, IL-6, IL-10, IL-18, ROS and MDA levels were increased and SOD activity was decreased in AAA and AAA + DSMO group versus sham group, and AAA + inhibitor could decrease IL-1β, IL-6, IL-10, IL-18, ROS and MDA levels, and increase SOD activity (Fig. 3). It is suggested that inflammatory factors level and oxidative stress could be decreased by ALOX5 inhibition.

### Effect of ALOX5 inhibition on cell pyroptosis and NF-κB pathway in AAA mice

**Fig. 4:**
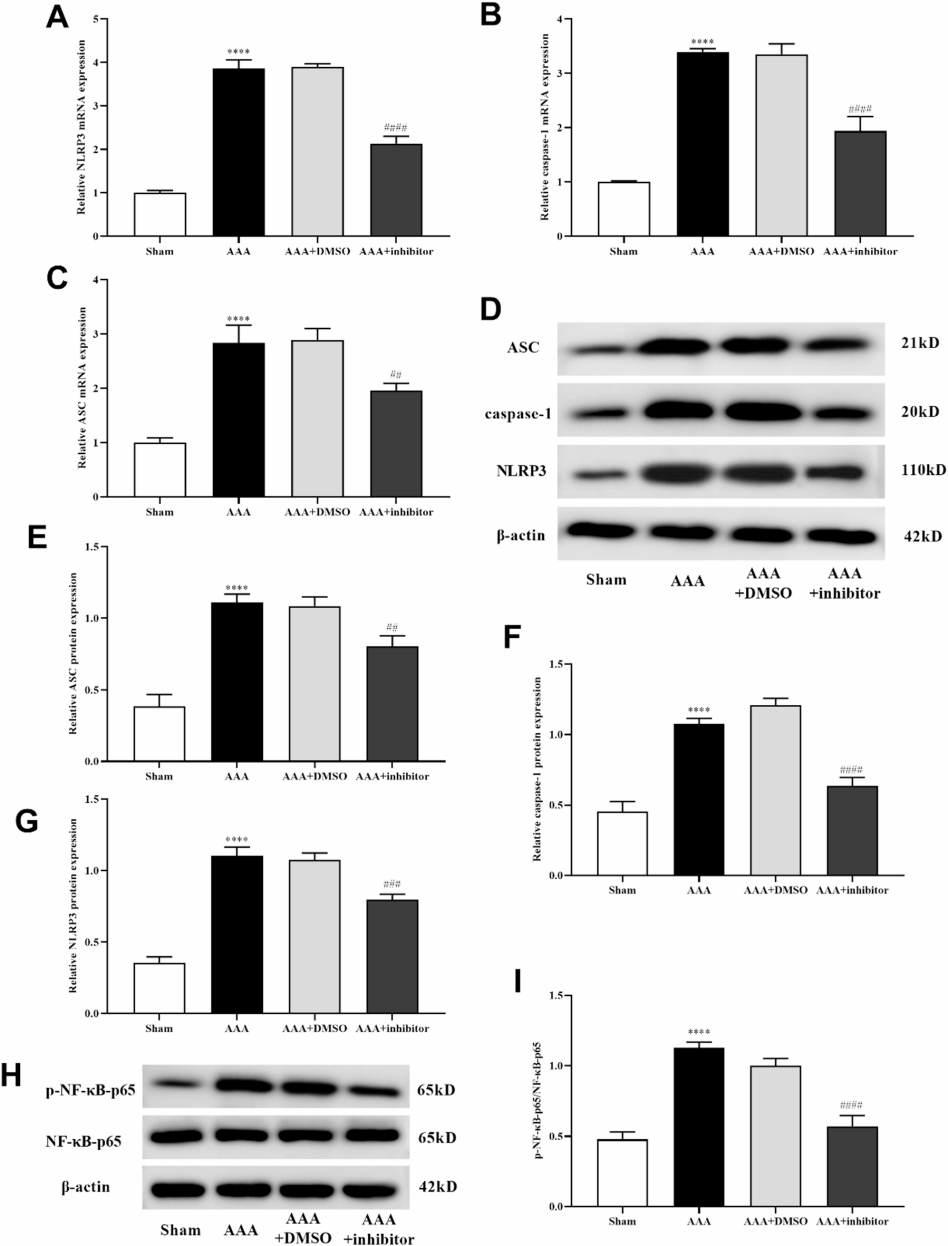
Effect of ALOX5 inhibition on cell pyroptosis and NF-κB pathway in AAA mice. (**A**, **B**, **C**, **D**, **E**, **F**,** G**) NLRP3, caspase-1, and ASC mRNA and protein expressions in each group. (**H**, **I**) Ang II-induced upregulation of p-NF-κB-p65 was blocked by ALOX5 inhibitor. Ang II: 1000 ng/kg/min. ALOX5 inhibitor: 30 mg/kg/day. ****P < 0.0001, compared with sham group. ##*P <* 0.01, ##*#P <* 0.001, ###*#P <* 0.0001, compared with AAA + DMSO group.

To investigate whether ALOX5 inhibition effect pyroptosis in AAA, pyroptosis related proteins (NLRP3, caspase-1, and ASC) expression were detected. NLRP3, caspase-1, and ASC protein expressions in AAA and AAA + DSMO group were increased versus those in sham group, while they were decreased after inhibitor addition (Fig. 4A-G). This suggested that ALOX5 associated with pyroptosis in AAA. Additionally, p-NF-κB-p65 proteins expression was increased in AAA and AAA + DSMO group versus control group, and ALOX5 inhibitor decreased its expression (Fig. 4H and I). This indicated that perhaps ALOX5 involved in the occurrence and development of AAA through NF-κB signaling.

### Ang II induced pyroptosis, oxidative stress and inflammatory response of MA-VSMCs

**Fig. 5:**
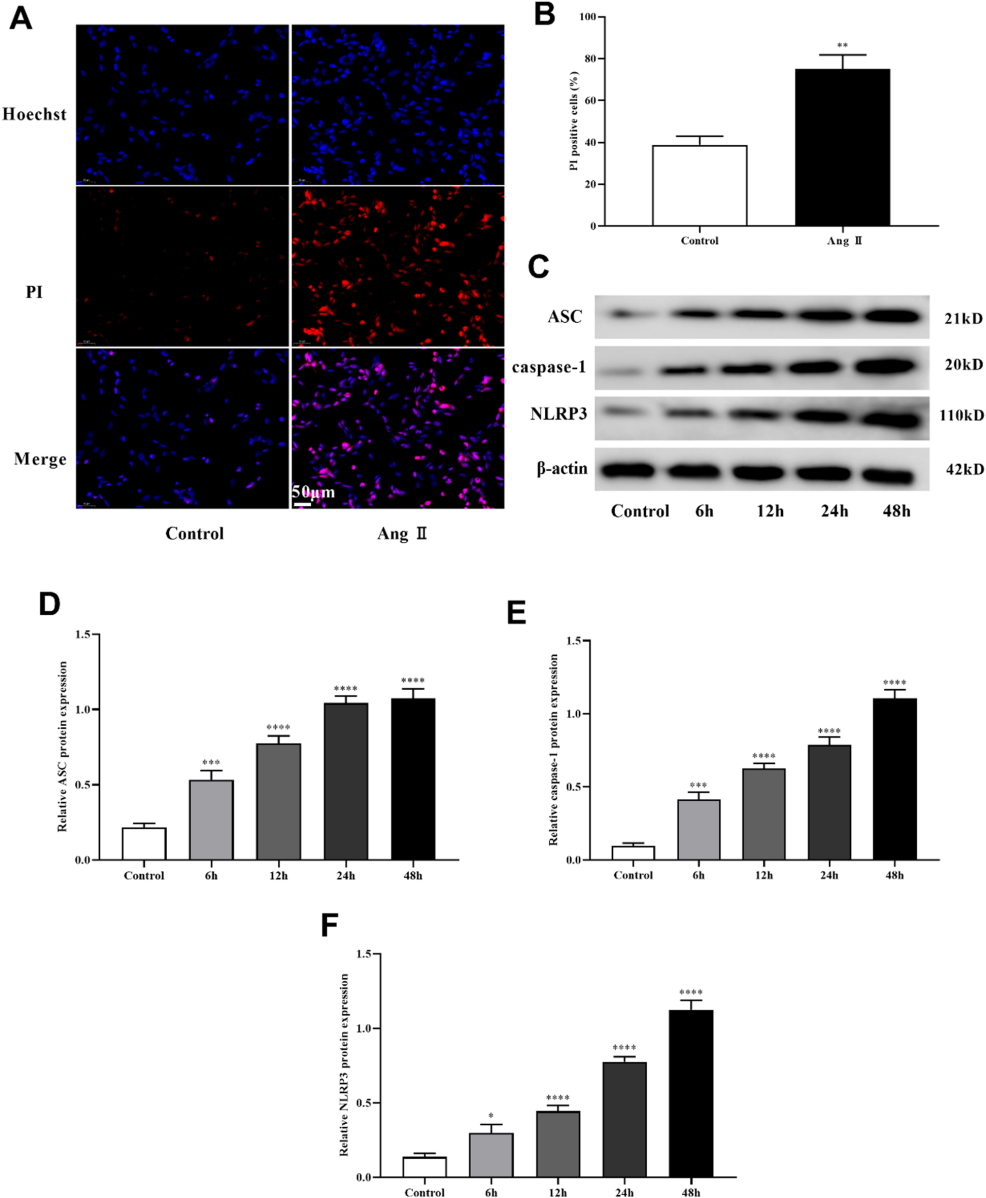
Ang II induced pyroptosis of MA-VSMCs. (**A**, **B**) Ratio of PI‑positive MA-VSMCs. PI is shown in red. (**C**, **D**, **E**, **F**) Expression of pyroptosis related proteins in MA-VSMCs. Ang II: 1 µmol/L. *P < 0.05, **P < 0.01, ***P < 0.001, ****P < 0.0001, compared with control group.

**Fig. 6:**
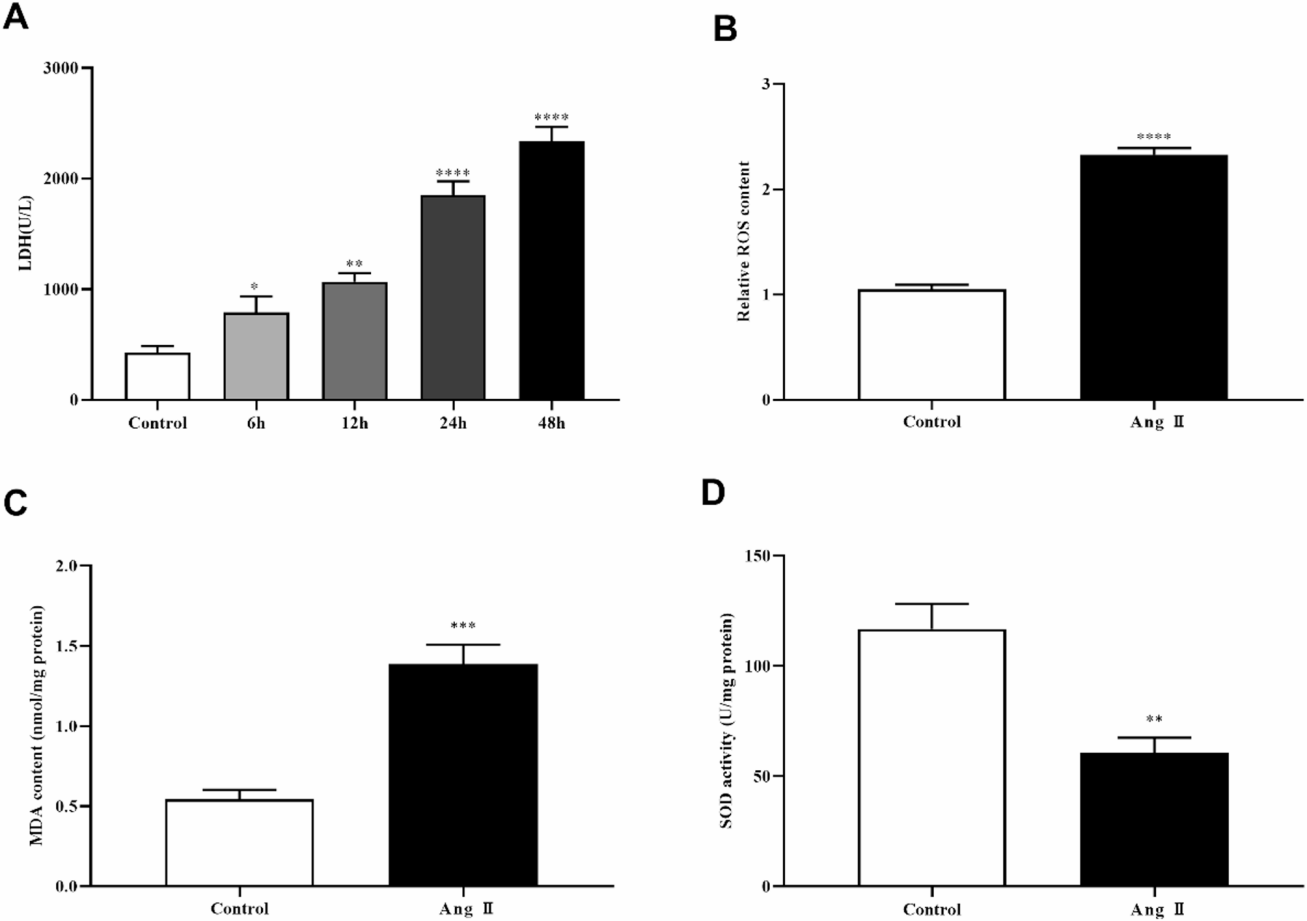
Effect of Ang II on LDH release and oxidative stress of MA-VSMCs. (**A**) LDH levels in the supernatant of MA-VSMCs stimulated with Ang II. (**B**, **C**, **D**) ROS and MDA levels, and SOD activity in MA-VSMCs following treatment with Ang II. Ang II: 1 µmol/L. *P < 0.05, **P < 0.01, ***P < 0.001, ****P < 0.0001, compared with control group.

**Fig. 7:**
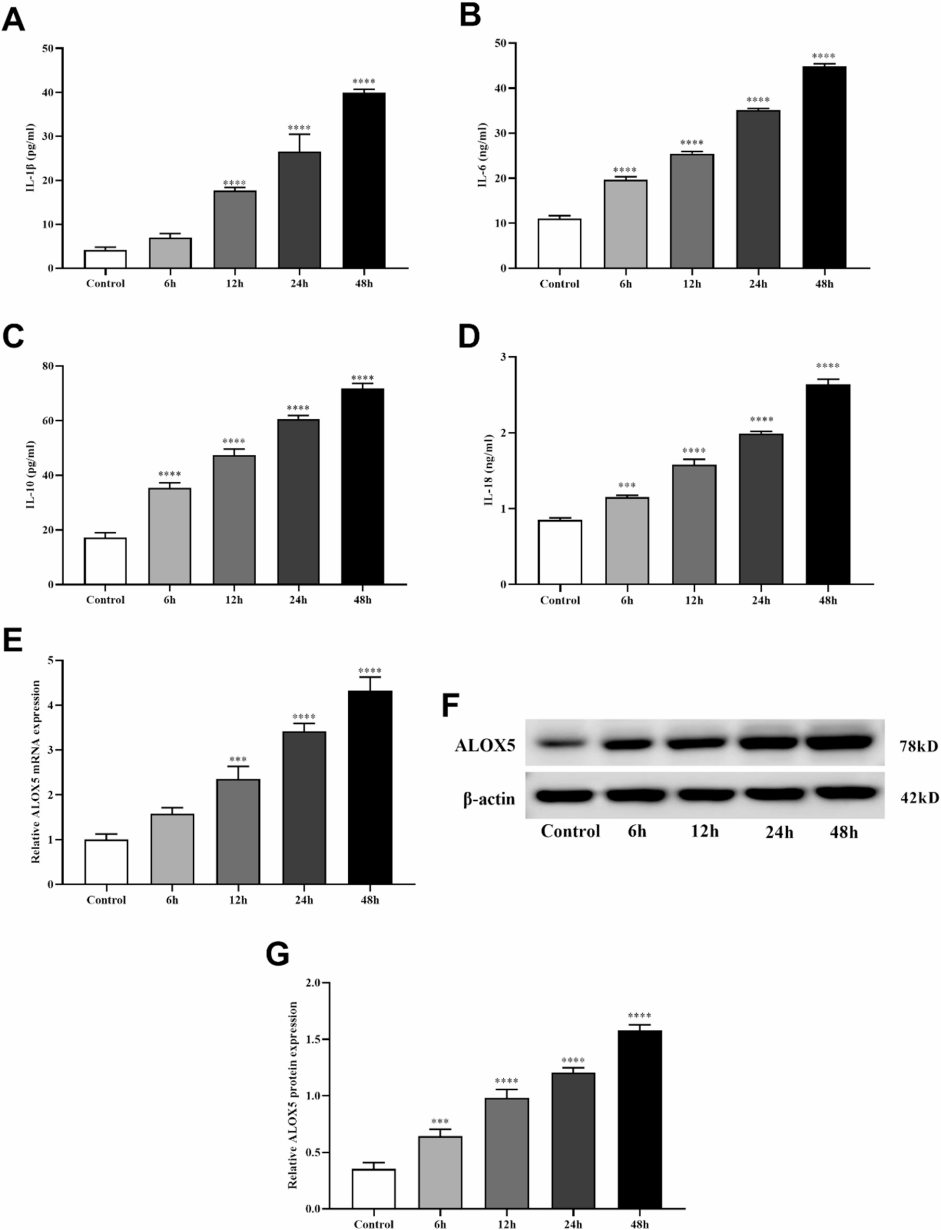
Effect of Ang II on inflammatory factors and ALOX5 expression of MA-VSMCs. (**A**, **B**, **C**, **D**) Levels of IL-1β, IL-6, IL-10 and IL-18 in MA-VSMCs after Ang II treatment. (**E**, **F**, **G**) ALOX5 expression in MA-VSMCs after Ang II treatment. Ang II: 1 µmol/L. ***P < 0.001, ****P < 0.0001, compared with control group.

To further verify the role of ALOX5 in AAA, we performed cell tests by treating MA-VSMCs with Ang II. Pyroptosis, levels of LDH, ROS, MDA and inflammatory factors, SOD activity and ALOX5 expression of MA-VSMCs were detected after Ang II treatment. The results showed that PI-positive cells, pyroptosis related proteins (NLRP3, caspase-1, and ASC) expression, LDH release, levels of ROS, MDA, IL-1β, IL-6, IL-10 and IL-18 of MA-VSMCs were increased and SOD activity of MA-VSMCs was decreased after Ang II treatment versus control (Figs. 5, 6 and 7A-D). Additionally, ALOX5 expression also increased in Ang II-stimulated MA-VSMCs (Fig. 7E-G).

### ALOX5 knockdown affected MA-VSMCs pyroptosis, oxidative stress and inflammatory response induced by Ang II

**Fig. 8:**
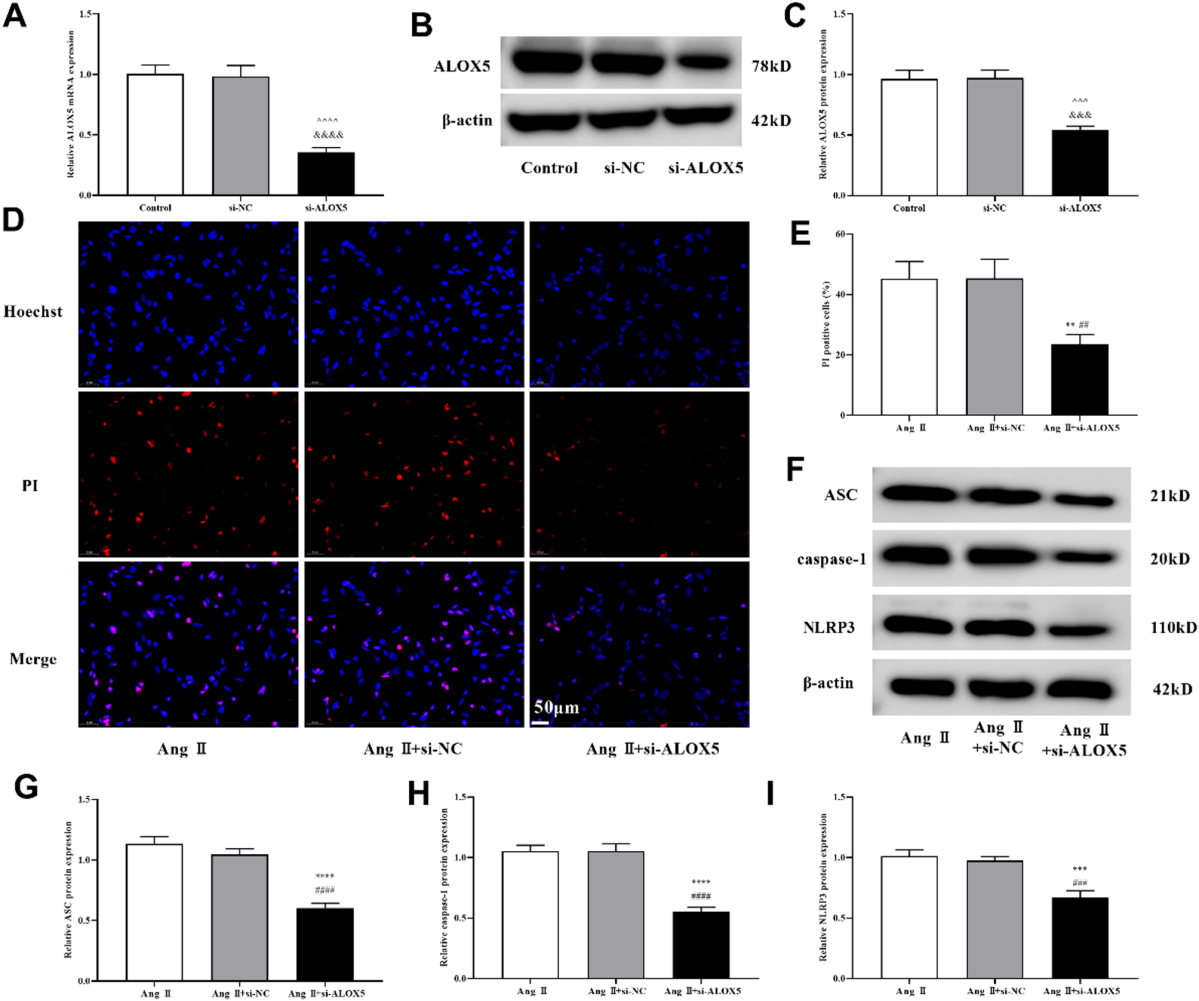
ALOX5 knockdown affected MA-VSMCs pyroptosis induced by Ang II. (**A**, **B**, **C**) Expression of ALOX5 mRNA and protein in MA-VSMCs with si-ALOX5 transfection. (**D**, **E**) Ratio of PI‑positive MA-VSMCs of each group after treatment. PI is shown in red. (**F**, **G**, **H**, **I**) Expression of pyroptosis related proteins in MA-VSMCs of each group after treatment. Ang II: 1 µmol/L. ^^^*P <* 0.001, ^^^^*P <* 0.0001, compared with control group. &&&*P <* 0.001, &&&&*P <* 0.0001, compared with si-NC group. **P < 0.01, ***P < 0.001, ****P < 0.0001, compared with Ang II group. ##*P <* 0.01, ###*P <* 0.001, ####*P <* 0.0001, compared with Ang II+si-NC group.

**Fig. 9:**
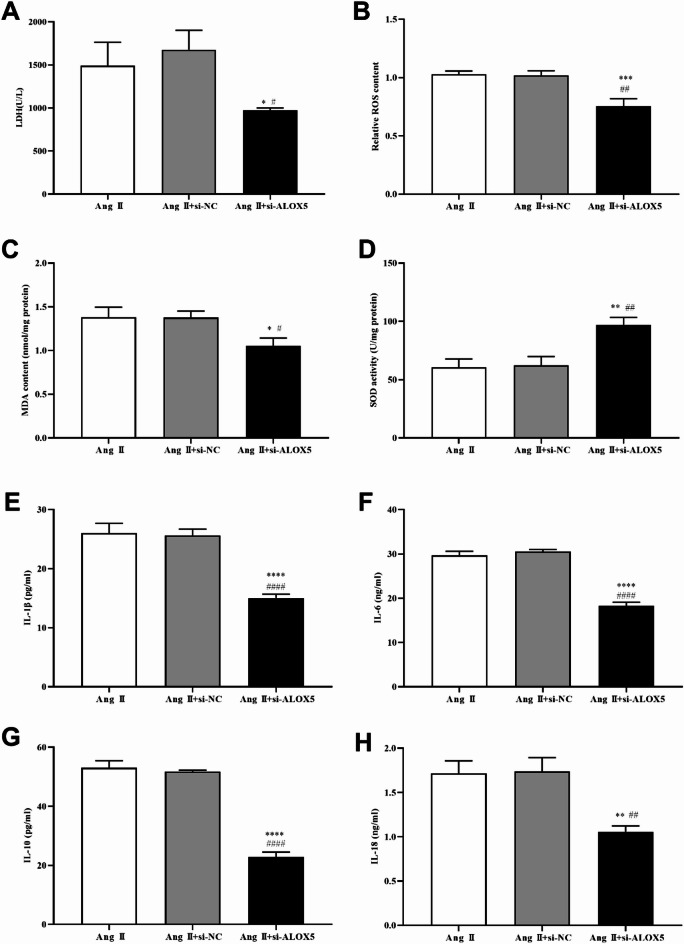
ALOX5 knockdown affected LDH release, oxidative stress and inflammatory factors of MA-VSMCs with Ang II treatment. (**A**) LDH levels in the supernatant of MA-VSMCs after treatment. (**B**, **C**, **D**) Levels of ROS and MDA, and SOD activity in MA-VSMCs after treatment. (**E**, **F**, **G**, **H**) Levels of IL-1β, IL-6, IL-10 and IL-18 in MA-VSMCs after treatment. Ang II: 1 µmol/L. *P < 0.05, **P < 0.01, ***P < 0.001, ****P < 0.0001, compared with Ang II group. #*P <* 0.05, ##*P <* 0.01, ####*P <* 0.0001, compared with Ang II+si-NC group.

To explore the underlying mechanisms of ALOX5 in AAA, we interfered with ALOX5 expression in MA-VASMCs by transfecting si-ALOX5. ALOX5 expression was decreased in si-ALOX5 group versus si-NC group (Fig. 8A-C). The above results indicated that ALOX5 expression was successfully silenced. Further experiments found that ALOX5 silencing inhibited the PI-positive cells, pyroptosis related proteins (NLRP3, caspase-1, and ASC) expression, release of LDH, ROS and MDA levels, and IL-1β, IL-6, IL-10 and IL-18 release induced by Ang II in MA-VSMCs, while it improved SOD activity (Figs. 8D- I and 9).

### ALOX5 effected MA-VSMCs pyroptosis by NF-κB pathway

**Fig. 10:**
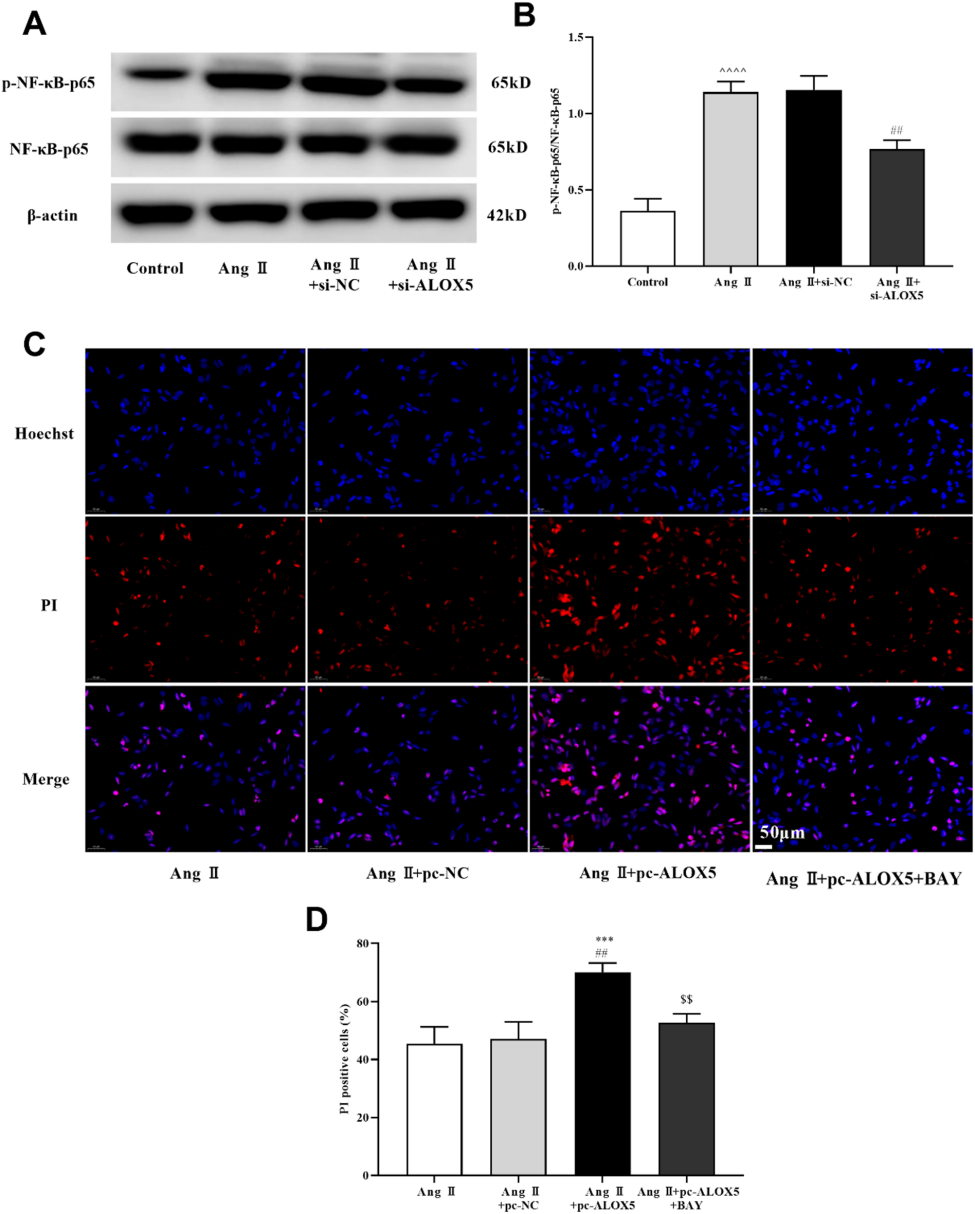
ALOX5 effected MA-VSMCs pyroptosis by NF-κB pathway. (**A**, **B**) Ang II-induced upregulation of p-NF-κB-p65 was blocked by ALOX5 knockdown. (**C**, **D**) Addition of BAY decreased the increasing of PI-positive cells in MA-VSMCs caused by ALOX5 overexpression and Ang II treatment. Ang II: 1 µmol/L. BAY: 1 µmol/L. ^^^^*P <* 0.0001, compared with control group. ***P < 0.001, compared with Ang II group. ##*P <* 0.01, compared with Ang II+si-NC or Ang II+pc-NC group. $$*P <* 0.01, compared with Ang II+pc-ALOX5 group.

The NF-κB pathway involves in pyroptosis, inflammation and apoptosis, and ALOX5 is reported to activate NF-κB pathway^37^. Results showed p-NF-κB-p65 proteins expression was increased in other groups versus control group, and ALOX5 silencing decreased its expression (Fig. 10A and B). This suggested that ALOX5 silencing inhibited Ang II-induced NF-κB pathway activation in MA-VSMCs.

**Fig. 11:**
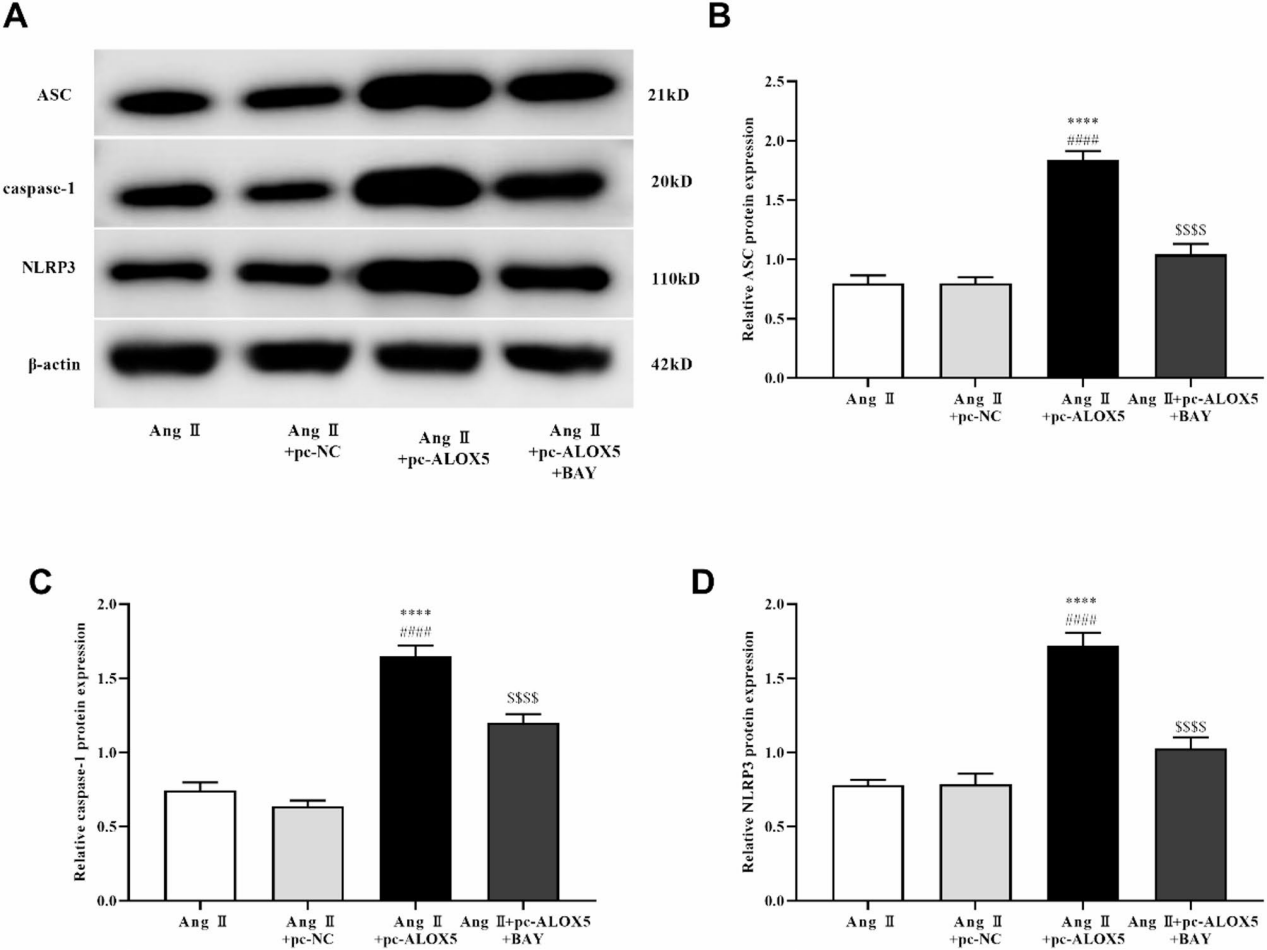
ALOX5 effected MA-VSMCs pyroptosis related protein expression by NF-κB pathway. (**A**, **B**, **C**, **D**) Addition of BAY decreased the increasing of pyroptosis related protein expression in MA-VSMCs caused by ALOX5 overexpression under Ang II treatment. Ang II: 1 µmol/L. BAY: 1 µmol/L. ****P < 0.0001, compared with Ang II group. ####*P <* 0.0001, compared with Ang II+pc-NC group. $$$$*P <* 0.0001, compared with Ang II+pc-ALOX5 group.

**Fig. 12:**
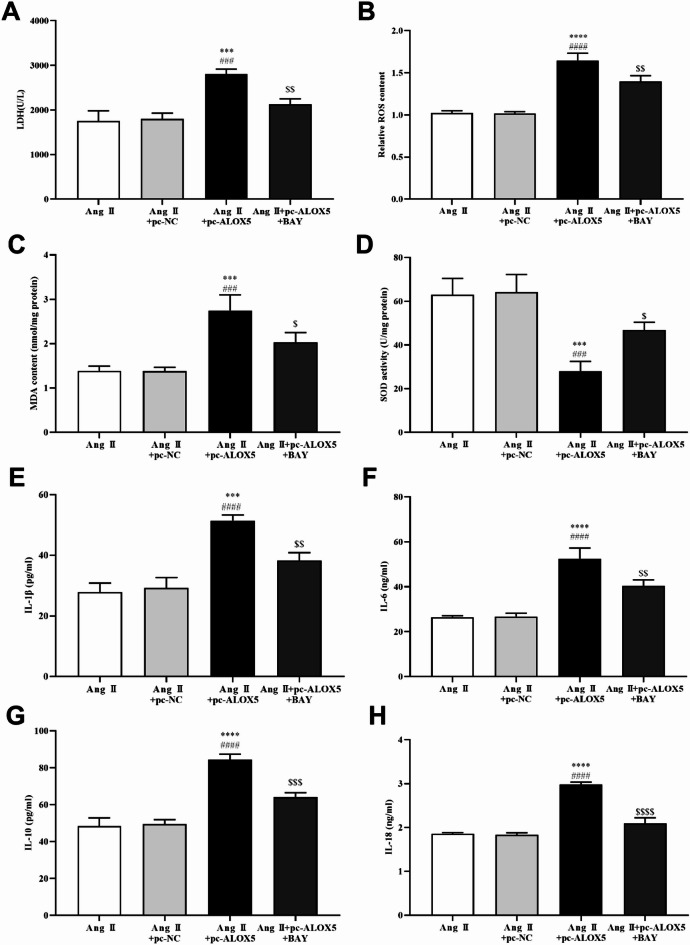
ALOX5 effected MA-VSMCs LDH release, oxidative stress and inflammatory cytokine levels by NF-κB pathway. (**A**) Addition of BAY decreased the LDH release in MA-VSMCs by ALOX5 overexpression and Ang II treatment. (**B**, **C**, **D**) Addition of BAY decreased the increasing of ROS and MDA levels and improved the inhibition of SOD activity in MA-VSMCs by ALOX5 overexpression and Ang II treatment. (**E**, **F**, **G**, **H**) Addition of BAY reversed the increasing of IL-1β, IL-6, IL-10 and IL-18 levels in MA-VSMCs caused by ALOX5 overexpression and Ang II treatment. Ang II: 1 µmol/L. BAY: 1 µmol/L. ***P < 0.001, ****P < 0.0001, compared with Ang II group. ###*P <* 0.001, ####*P <* 0.0001, compared with Ang II+pc-NC group. $*P <* 0.05, $$*P <* 0.01, $$$*P <* 0.001, $$$$*P <* 0.0001, compared with Ang II+pc-ALOX5 group.

In order to confirm that ALOX5 regulates pyroptosis through NF-κB pathway in MA-VSMCs, BAY11-7082 (BAY, an inhibitor of the NF-κB pathway) was added while ALOX5 was overexpressed. ALOX5 overexpression promoted the PI-positive cells, pyroptosis related proteins (NLRP3, caspase-1, and ASC) expression, LDH release, ROS and MDA levels, and IL-1β, IL-6, IL-10 and IL-18 release induced by Ang II in MA-VSMCs, while they were reversed by the addition of BAY (Figs. 10C and D, 11 and 12). Moreover, ALOX5 overexpression inhibited SOD activity of MA-VSMCs after Ang II treatment, while it was reversed by the addition of BAY (Fig. 12D).

## Discussion

AAA is a common and potentially fatal vascular aneurysm, which is induced by high-risk cardiovascular factors including hypertension, hyperlipidemia, and atherosclerosis^38^. AAA incidence has been increasing year by year and has become one of the killers threatening the health of the elderly in recent years^39^. Clinically, surgical treatment and conventional drug treatment of AAA can improve its clinical symptoms, but the incidence of rapid death of patients due to ineffective rescue caused by AAA rupture has remained high^40^. Therefore, it has great clinical significance to clarify the pathogenesis of AAA to reduce AAA incidence at the source.

AAA formation is closely related to cell pyroptosis, which involves in AAA progression by promoting inflammatory cell infiltration and leading to vascular wall elastic layer destruction^41^. Regulated cell death of VSMCs, including pyroptosis, relates to aortic aneurysm occurrence and development^42^. ALOX5 is a key enzyme that triggers lipid peroxidation, which stimulates inflammatory responses and induces various cell death modes including ferroptosis, pyroptosis, and apoptosis^30,43^. To this end, this study induced ApoE^−/−^ mouse AAA model with Ang II and clarified the ALOX5 expression. ALOX5 expression in abdominal aorta tissue of AAA mice was significantly increased. Ang II induced group of mice had thickening of the abdominal aortic vascular wall, inflammatory cell infiltration, rupture and destruction of elastic fibers, and pathological increase in collagen fibers of the blood vessels, and the diameter of the abdominal aorta significantly increased, indicating the successful construction of the AAA mouse model. After the addition of inhibitors, the above-mentioned pathological changes of AAA tissues were alleviated. Moreover, levels of inflammatory factors and oxidative stress, and pyroptosis were decreased in in mice AAA after inhibitors addition. Those results suggested that ALOX5 may be one potential target for AAA treatment. Therefore, it has great significance to use ALOX5 as the entry point for AAA research.

Pyroptosis-related proteins were expressed in AAA tissues of AAA mouse. Ang II-induced pyroptosis in ApoE^−/−^ mouse AAA was inhibited by ALOX5 inhibitor. Therefore, the present study speculated that ALOX5 may promote AAA formation by regulating pyroptosis of VSMCs. To clarify the effect of ALOX5 on pyroptosis of AAA VSMCs, further experiments were performed by knocking down ALOX5 expression in MA-VSMCs. After treating MA-VSMCs with Ang II, the levels of PI-positive cells and pyroptosis related proteins expression were detected, and it was found that they were increased versus control group. The above results indicated that the occurrence of cell pyroptosis was exacerbated in MA-VSMCs induced by Ang II. At the same time, LDH release, oxidative stress, and inflammatory factors were also increased after Ang II induction. ALOX5 expression in MA-VSMCs induced by Ang II was increased, and PI-positive cells, pyroptosis related protein expression, LDH release, oxidative stress and inflammatory factors could be improved after ALOX5 knockdown, suggesting that ALOX5 plays a promoting role in Ang II-induced pyroptosis of MA-VSMCs.

NF-κB is a transcription factor in the cell nucleus that regulates inflammation and cellular immunity and may be involved in AAA occurrence and development^44^. Here, WB experiment was conducted to explore NF-κB pathway changes after Ang II intervention. It was found that the phosphorylated NF-κB-p65 increased in mice and MA-VSMCs after Ang II treatment, indicating NF-κB pathway was activated. Phosphorylated NF-κB-p65 in MA-VSMCs with ALOX5 knockdown and Ang II intervention was reduced. This means that knocking down ALOX5 inhibited NF-κB pathway activation. Further studies showed that the addition of NF-κB pathway inhibitor BAY could reverse the promoting effect of ALOX5 overexpression on the PI-positive cells, pyroptosis related proteins expression, release of LDH, oxidative stress and inflammatory cytokines levels in Ang II-induced MA-VSMCs. This suggested that ALOX5 could regulate Ang II-induced pyroptosis of MA-VSMCs through NF-κB pathway, thereby promoting AAA progression.

## Conclusion

In summary, VSMCs pyroptosis can be inhibited by inhibiting ALOX5 and NF-κB pathway activation, thereby improving the progression of AAA. ALOX5 may act as a potential target for AAA treatment. This study provides new directions and ideas for the treatment of AAA. However, this study also has certain limitations. For example, this study only focused on the ALOX5 and NF-κB pathways, and did not fully explore other possible mechanisms and targets. There may be other unknown key factors that play vital role of AAA development. Moreover, mechanism of ALOX5 and NF-κB pathways involved in AAA progression needs to be further studied in the future.

## Supplementary Information

Below is the link to the electronic supplementary material.

Supplementary Material 1

## Data Availability

The dataset that support the results and findings of this research are available from the corresponding author on reasonable request.

## References

[1] Zhao, Y. et al. Colchicine protects against the development of experimental abdominal aortic aneurysm. *Clin. Sci. (London England: 1979)*. **137** (19), 1533–1545 (2023). 10.1042/CS20230499 PMC10550771 37748024

[2] Hosseini, A. et al. Effect of Statins on abdominal aortic aneurysm. *Eur. J. Pharm. Sci.* **178**, 106284 (2022). 36038100 10.1016/j.ejps.2022.106284

[3] Puertas-Umbert, L. et al. Novel Pharmacological approaches in abdominal aortic aneurysm. *Clin. Sci.* **137** (15), 1167–1194 (2023). 10.1042/CS20220795 PMC10415166 37559446

[4] Gao, J. et al. The mechanism and therapy of aortic aneurysms. *Signal. Transduct. Target. Therapy*. **8** (1), 55 (2023). 10.1038/s41392-023-01325-7 PMC9898314 36737432

[5] Chen, A. J. et al. Frailty among veterans undergoing abdominal aortic aneurysm repair. *Ann. Vasc. Surg.* **92**, 18–23 (2023). 36690250 10.1016/j.avsg.2023.01.007

[6] Chen, J. & Sheng, Y. Outcomes of abdominal aortic aneurysm repairs: endovascular aneurysm vs open surgical repairs. *Asian J. Surg.* **44** (11), 1492–1492 (2021). 34588137 10.1016/j.asjsur.2021.08.025

[7] Chen, H. Z. et al. Age-associated Sirtuin 1 reduction in vascular smooth muscle links vascular senescence and inflammation to abdominal aortic aneurysm. *Circul. Res.* **119** (10), 1076–1088 (2016). 10.1161/CIRCRESAHA.116.308895 PMC6546422 27650558

[8] Shah, P. K. Inflammation, metalloproteinases, and increased proteolysis: an emerging pathophysiological paradigm in aortic aneurysm. *Circulation* **96** (7), 2115–2117 (1997). 9337176 10.1161/01.cir.96.7.2115

[9] Sánchez-Infantes, D. et al. Oxidative stress and inflammatory markers in abdominal aortic aneurysm. *Antioxidants* **10** (4), 602 (2021). 33919749 10.3390/antiox10040602 PMC8070751

[10] McCormick, M. L., Gavrila, D. & Weintraub, N. L. Role of oxidative stress in the pathogenesis of abdominal aortic aneurysms. *Arterioscler. Thromb. Vasc. Biol.* **27**(3), 461–469 (2007). 10.1161/01.ATV.0000257552.94483.14 17218601

[11] Yuan, Z. et al. Abdominal aortic aneurysm: roles of inflammatory cells. *Front. Immunol.* **11**, 609161 (2021). 33613530 10.3389/fimmu.2020.609161 PMC7886696

[12] Pi, S. et al. The role of inflammasome in abdominal aortic aneurysm and its potential drugs. *Int. J. Mol. Sci.* **25** (9), 5001 (2024). 38732221 10.3390/ijms25095001 PMC11084561

[13] Zhu, J. et al. Inflammation in abdominal aortic aneurysm: cause or co-morbidity? *Can. J. Cardiol.* **40** (12), 2378–2391 (2024). 39181326 10.1016/j.cjca.2024.08.274

[14] Guzik, B. et al. Mechanisms of oxidative stress in human aortic aneurysms—association with clinical risk factors for atherosclerosis and disease severity. *Int. J. Cardiol.* **168** (3), 2389–2396 (2013). 23506637 10.1016/j.ijcard.2013.01.278 PMC3819986

[15] Papalambros, E. et al. Malondialdehyde as an indicator of oxidative stress during abdominal aortic aneurysm repair. *Angiology* **58** (4), 477–482 (2007). 17875961 10.1177/0003319707305246

[16] Yu, Z. et al. Endogenous superoxide dismutase activation by oral administration of riboflavin reduces abdominal aortic aneurysm formation in rats. *J. Vasc. Surg.* **64** (3), 737–745 (2016). 26070605 10.1016/j.jvs.2015.03.045

[17] Yang, F. et al. Pyroptosis and pyroptosis-inducing cancer drugs. *Acta Pharmacol. Sin.* **43** (10), 2462–2473 (2022). 35288674 10.1038/s41401-022-00887-6 PMC9525650

[18] Yarovinsky, T. O. et al. Pyroptosis in cardiovascular diseases: pumping gasdermin on the fire. *Semin. Immunol.* **69**, 101809 (2023). 37478801 10.1016/j.smim.2023.101809 PMC10528349

[19] Wei, Y. et al. Pyroptosis-induced inflammation and tissue damage. *J. Mol. Biol.* **434** (4), 167301 (2022). 34653436 10.1016/j.jmb.2021.167301 PMC8844146

[20] Zhou, J. et al. Pyroptosis and degenerative diseases of the elderly. *Cell Death Dis.* **14** (2), 94 (2023). 36755014 10.1038/s41419-023-05634-1 PMC9908978

[21] Yu, P. et al. Pyroptosis: mechanisms and diseases. *Signal. Transduct. Target. Ther.* **6**(1), 128 (2021). 10.1038/s41392-021-00507-5 PMC8005494 33776057

[22] Samir, P., Malireddi, R. S. & Kanneganti, T. D. The panoptosome: a deadly protein complex driving pyroptosis, apoptosis, and necroptosis (PANoptosis). *Front. Cell. Infect. Microbiol.* **10**, 238 (2020). 10.3389/fcimb.2020.00238 PMC7283380 32582562

[23] Wang, Q. et al. Pyroptosis: a pro-inflammatory type of cell death in cardiovascular disease. *Clin. Chim. Acta*. **510**, 62–72 (2020). 32622968 10.1016/j.cca.2020.06.044

[24] Zhao, S. et al. Integrated analysis of bulk RNA-seq and single-cell RNA-seq reveals the function of pyrocytosis in the pathogenesis of abdominal aortic aneurysm. *Aging (Albany NY)*. **15** (24), 15287 (2023). 38112597 10.18632/aging.205350 PMC10781497

[25] Lu, H. et al. Vascular smooth muscle cells in aortic aneurysm: from genetics to mechanisms. *J. Am. Heart Association*. **10** (24), e023601 (2021). 10.1161/JAHA.121.023601 PMC9075263 34796717

[26] Qian, G. et al. Abdominal aortic aneurysm formation with a focus on vascular smooth muscle cells. *Life* **12** (2), 191 (2022). 35207478 10.3390/life12020191 PMC8880357

[27] Chen, L. et al. Research progress of effect of smooth muscle cell pyroptosis on relevant diseases. *Guangxi Med. J.* **43** (14), 1749–1753 (2021).

[28] Di, C. et al. Pyroptosis of vascular smooth muscle cells as a potential new target for preventing vascular diseases. *Cardiovasc. Drugs Ther.* 1–12 (2024). 10.1007/s10557-024-07578-w 38822974

[29] Yin, Z. et al. Regulated vascular smooth muscle cell death in vascular diseases. *Cell Prolif.* **57** (11), e13688 (2024). 10.1111/cpr.13688 PMC11533065 38873710

[30] Gaschler, M. M. & Stockwell, B. R. Lipid peroxidation in cell death. *Biochem. Biophys. Res. Commun.* **482** (3), 419–425 (2017). 28212725 10.1016/j.bbrc.2016.10.086 PMC5319403

[31] He, J. et al. Association analysis of ALOX5 gene polymorphisms with stroke risk: a case-control study in a Chinese Han population. *Int. J. Clin. Exp. Pathol.* **9** (4), 4432–4437 (2016).

[32] Back, M. Inhibitors of the 5-lipoxygenase pathway in atherosclerosis. *Curr. Pharm. Design*. **15** (27), 3116–3132 (2009). 10.2174/138161209789058020 19754386

[33] Visvikis-Siest, S. & Marteau, J. B. Genetic variants predisposing to cardiovascular disease. *Curr. Opin. Lipidol.* **17** (2), 139–151 (2006). 16531750 10.1097/01.mol.0000217895.67444.de

[34] Araujo, N. N. F. et al. Dysregulation of MicroRNAs and target genes networks in human abdominal aortic aneurysm tissues. *PLoS One*. **14** (9), e0222782 (2019). 31539405 10.1371/journal.pone.0222782 PMC6754147

[35] Bhamidipati, C. M. et al. 5-Lipoxygenase pathway in experimental abdominal aortic aneurysms. *Arterioscler. Thromb. Vasc. Biol.* **34**(12), 2669–2678 (2014). 10.1161/ATVBAHA.114.304016 PMC4239157 25324573

[36] Liao, F. et al. Disulfiram protects against abdominal aortic aneurysm by ameliorating vascular smooth muscle cells pyroptosis. *Cardiovasc. Drugs Ther.* **37** (6), 1–14 (2023). 35723784 10.1007/s10557-022-07352-w

[37] Zhao, Y. et al. Lipid metabolism enzyme 5-LOX and its metabolite LTB4 are capable of activating transcription factor NF-κB in hepatoma cells. *Biochem. Biophys. Res. Commun.* **418** (4), 647–651 (2012). 22293202 10.1016/j.bbrc.2012.01.068

[38] Haque, K. & Bhargava, P. Abdominal aortic aneurysm. *Am. Family Phys.* **106** (2), 165–172 (2022). 35977132

[39] Wu, J. Q., Wang, W. & Zheng, Y. H. Role of vascular aging in the pathogenesis of abdominal aortic aneurysm and potential therapeutic targets. *Acta academiae medicinae sinicae* **43**(6), 962–968 (2021). 10.3881/j.issn.1000-503X.13453 34980338

[40] Mangum, K., Gallagher, K. & Davis, F. M. The role of epigenetic modifications in abdominal aortic aneurysm pathogenesis. *Biomolecules* **12** (2), 172 (2022). 35204673 10.3390/biom12020172 PMC8961599

[41] Yao, Y. et al. Effect of pyroptosis in the formation of angiotensin II-induced abdominal aortic aneurysm. *Practical J. Cardiac Cereb. Pneumal Vascular Disease*. **30** (5), 73–79 (2022).

[42] Han, X., Liu, X. & Yang, B. The role of vascular smooth muscle cell regulatory death in the occurrence and development of aortic aneurysm. *Chin. J. Geriatric Heart Brain Vessel Dis.* **26** (03), 352–354 (2024).

[43] Zhao, Q. et al. Role of ALOX5 in non-small cell lung cancer: A potential therapeutic target associated with immune cell infiltration. *Zhong Nan Da Xue Xue Bao Yi Xue Ban*. **48** (3), 311–322 (2023). 37164914 10.11817/j.issn.1672-7347.2023.220427 PMC10930070

[44] Chen, S. et al. Paeonol ameliorates abdominal aortic aneurysm progression by the NF-κB pathway. *Ann. Vasc. Surg.* **77**, 255–262 (2021). 34411666 10.1016/j.avsg.2021.06.003

