# Supplementary material for: ALOX5 regulates vascular smooth muscle cells pyroptosis to affect abdominal aortic aneurysm formation
Source: Sci Rep. 2025 Aug 9;15:29123. doi: 10.1038/s41598-025-14268-6 (PMC12334731; doi:10.1038/s41598-025-14268-6)
Supplement: Supplementary file 1 — Supplementary Material 1 [file 41598_2025_14268_MOESM1_ESM.docx]

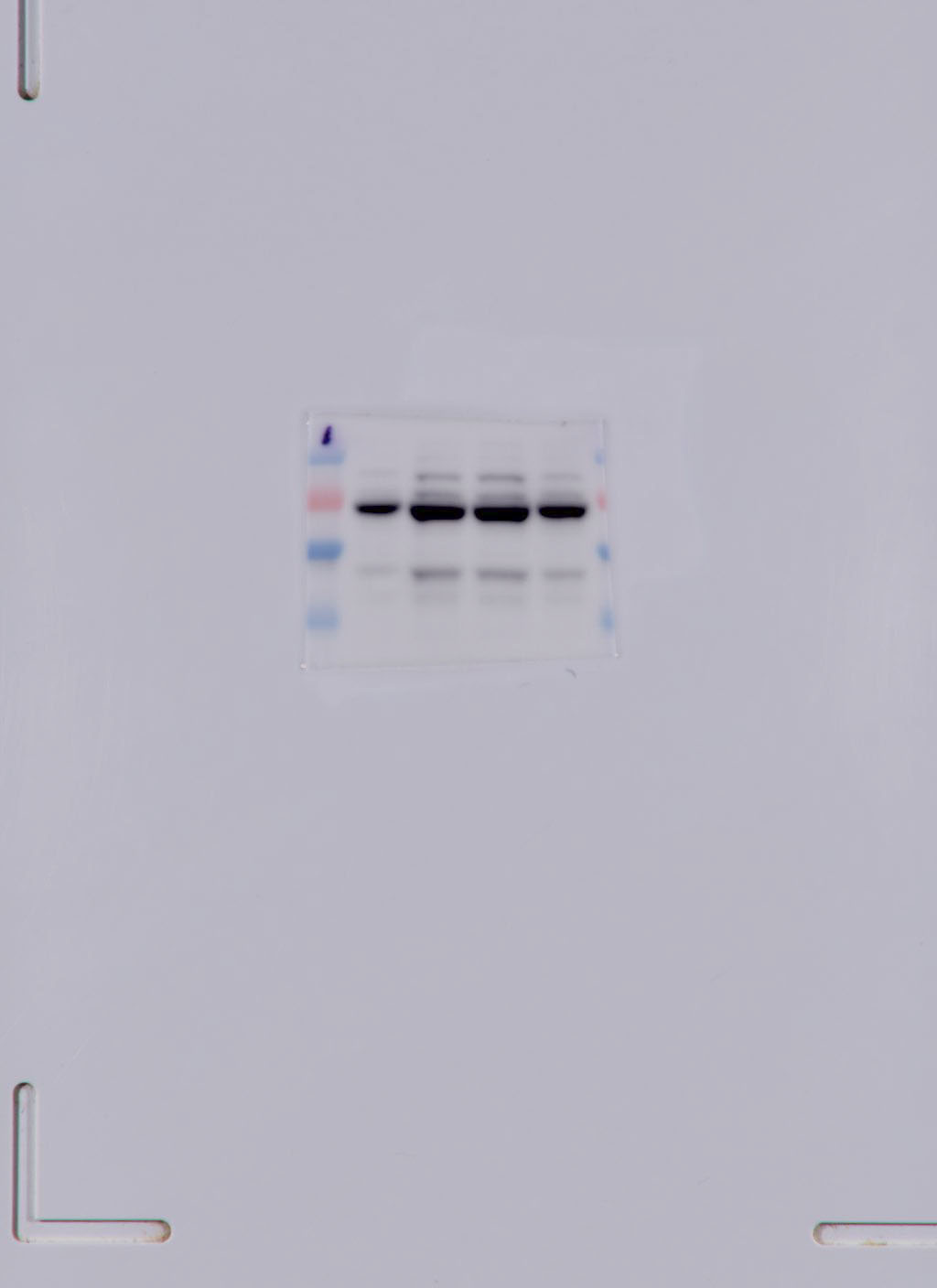


ALOX5

Besides marker in the picture, from left to right are the Sham, AAA, AAA+DMSO, and AAA+inhibitor groups in sequence.


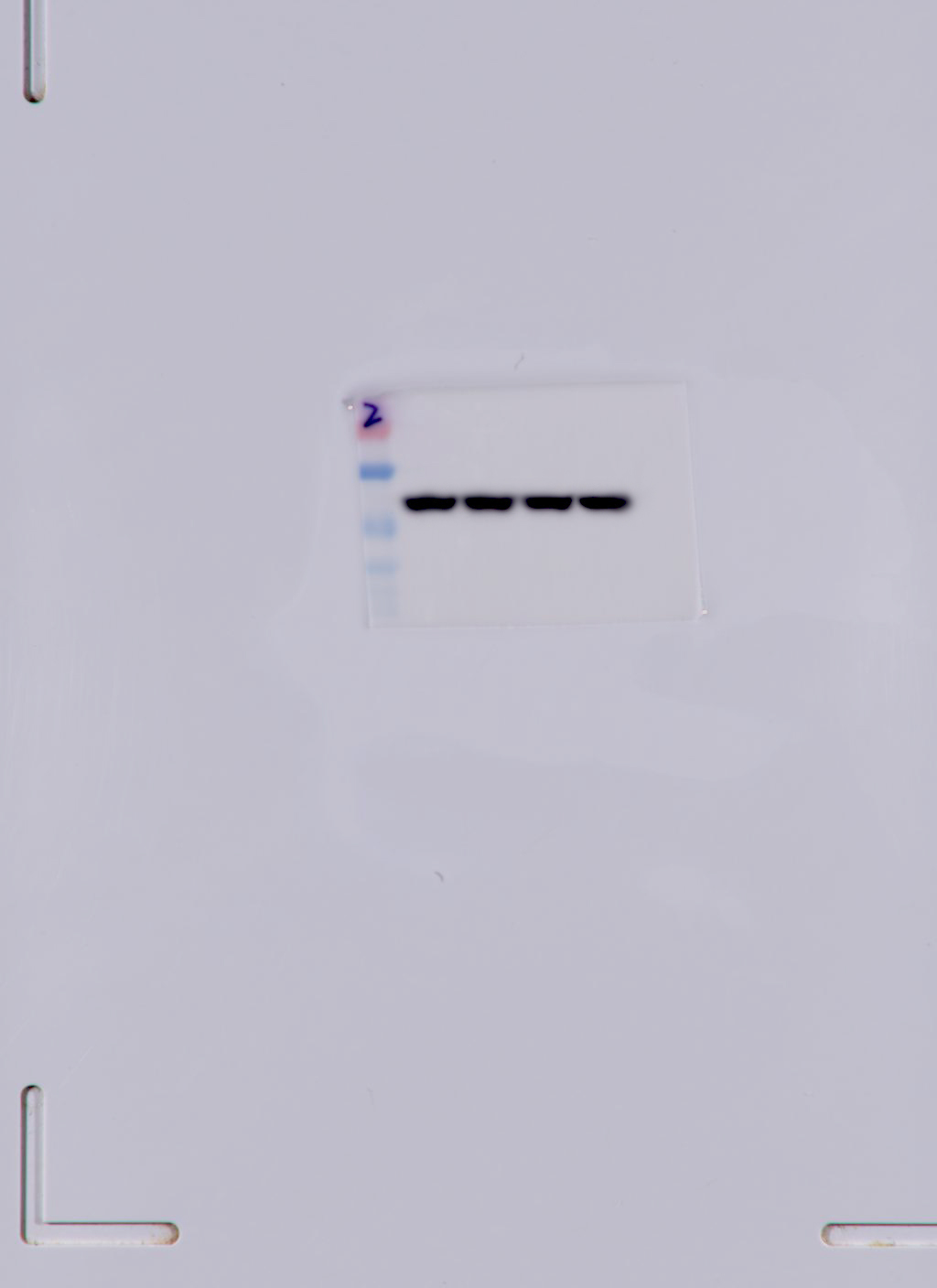


β-actin

Besides marker in the picture, from left to right are the Sham, AAA, AAA+DMSO, and AAA+inhibitor groups in sequence.

Figure 1A


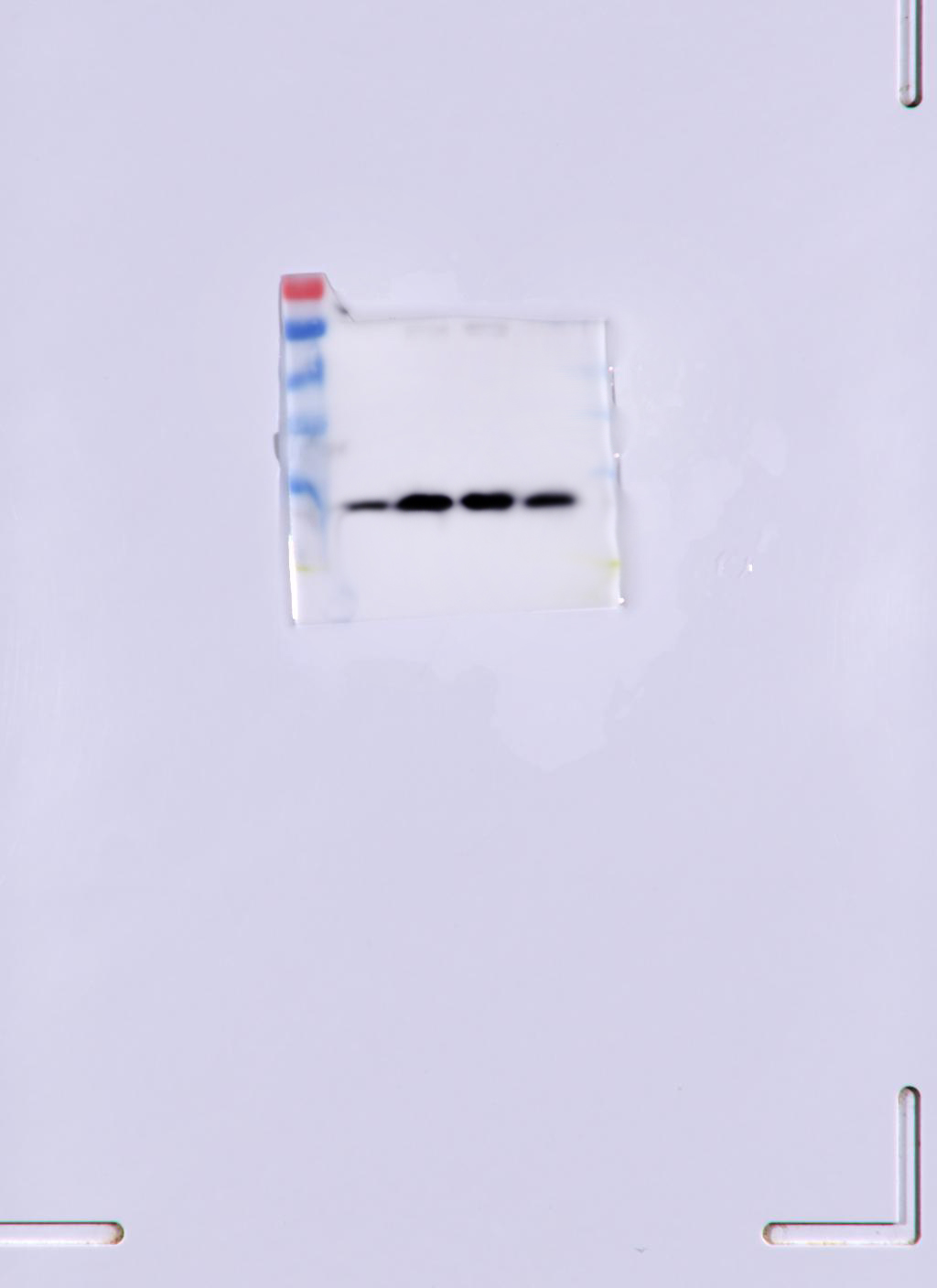


ASC

Besides marker in the picture, from left to right are the Sham, AAA, AAA+DMSO, and AAA+inhibitor groups in sequence.


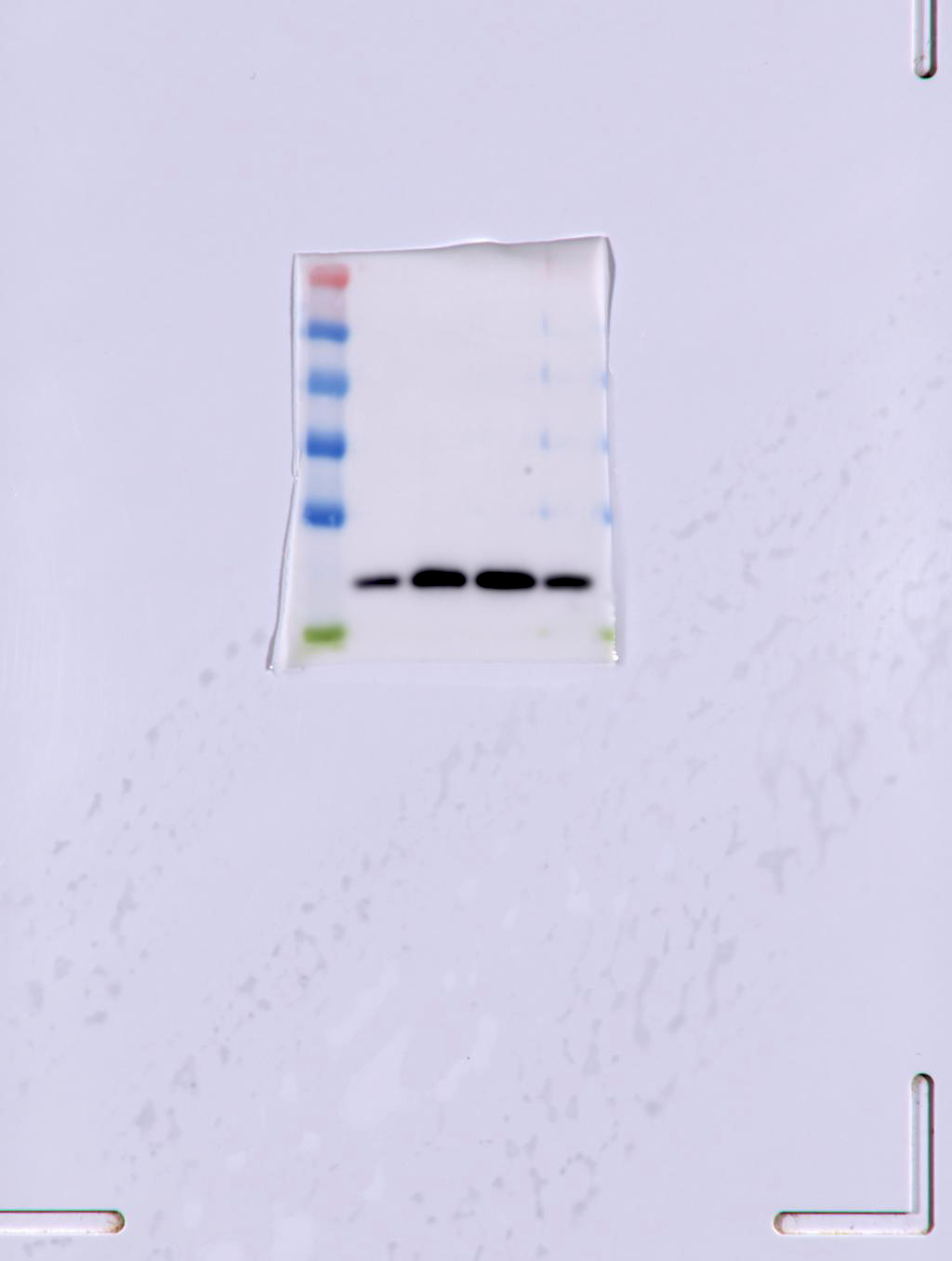


caspase-1

Besides marker in the picture, from left to right are the Sham, AAA, AAA+DMSO, and AAA+inhibitor groups in sequence.


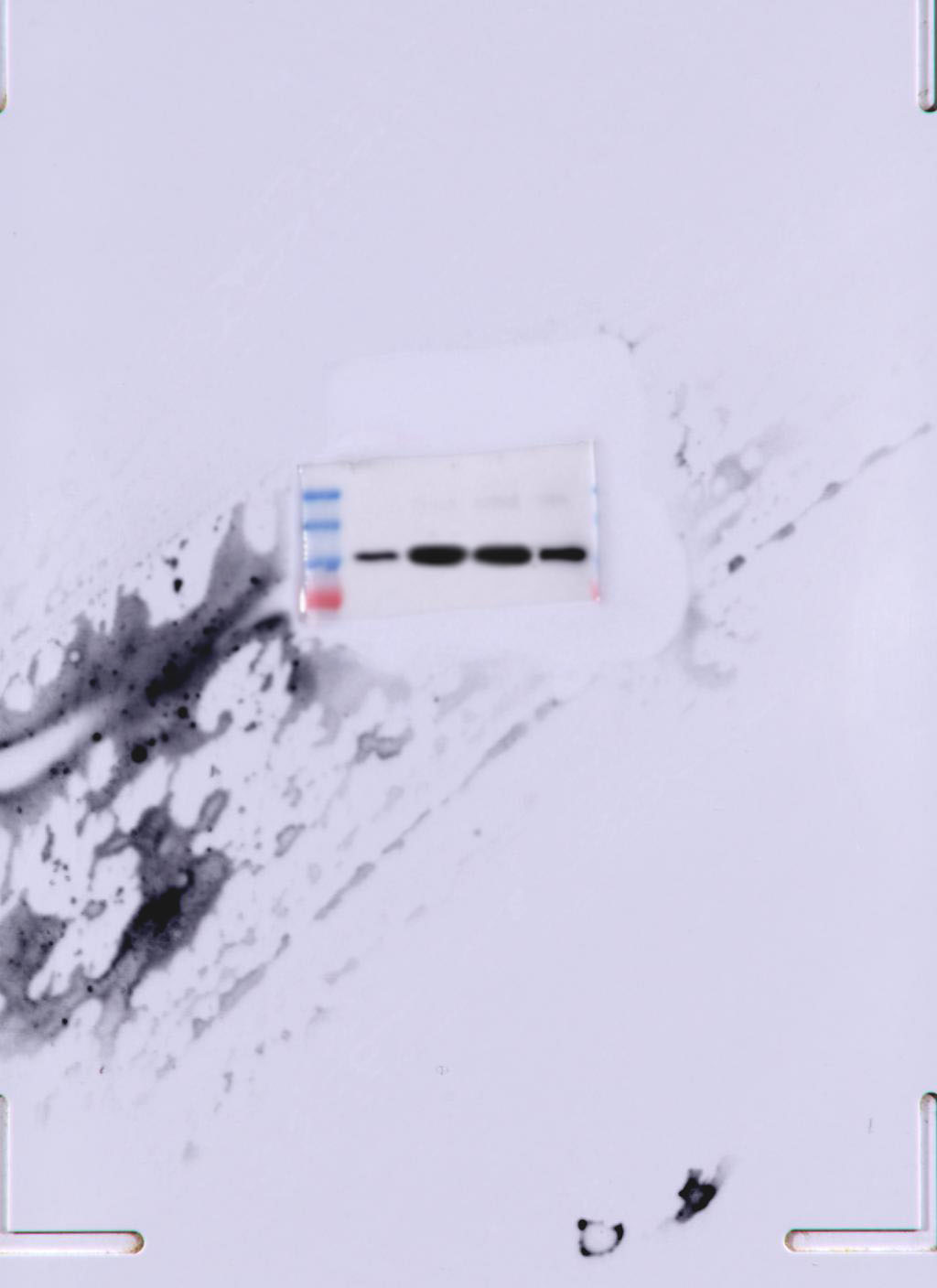


NLRP3

Besides marker in the picture, from left to right are the Sham, AAA, AAA+DMSO, and AAA+inhibitor groups in sequence.


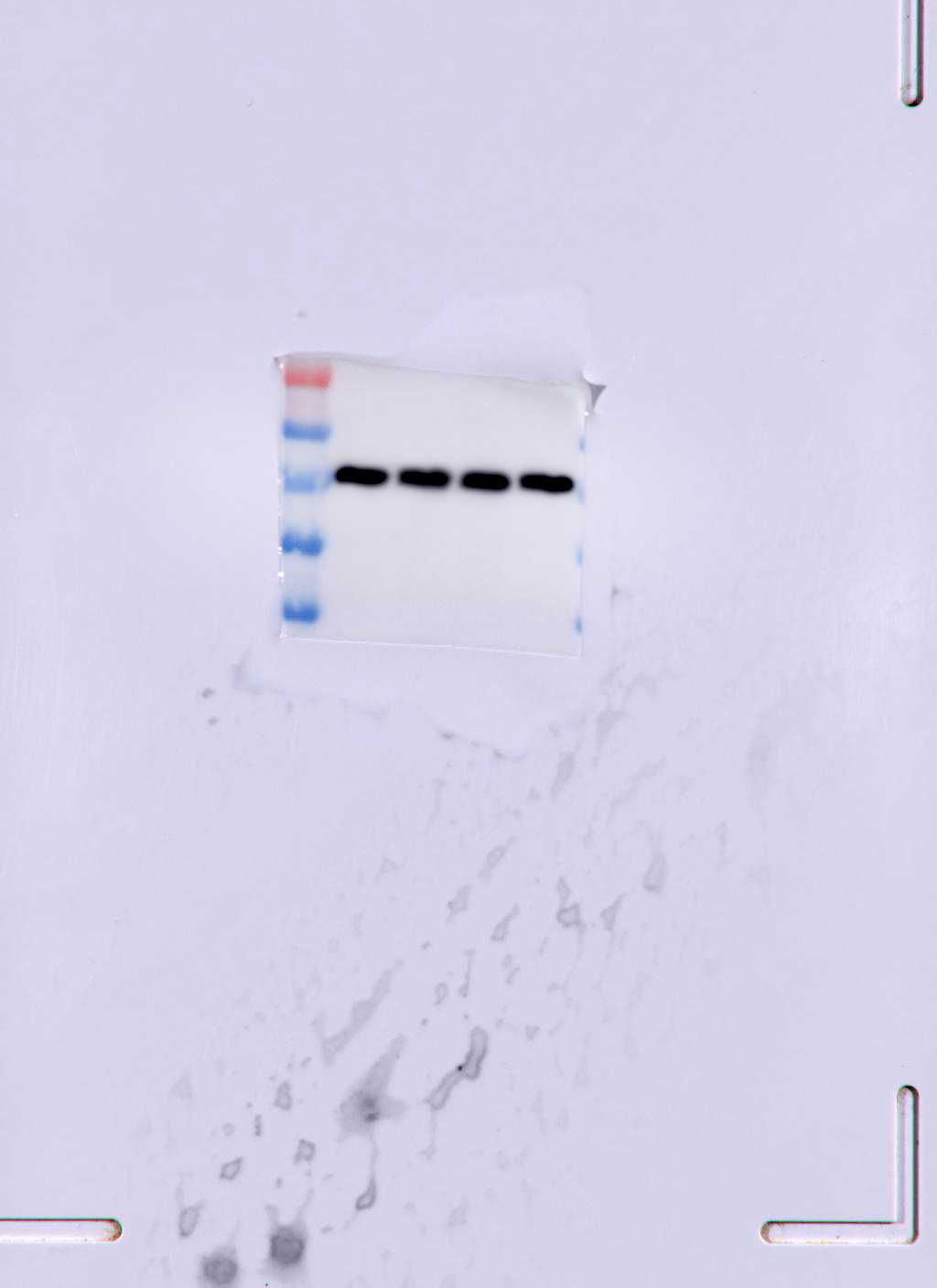


β-actin

Besides marker in the picture, from left to right are the Sham, AAA, AAA+DMSO, and AAA+inhibitor groups in sequence.

Figure 4D


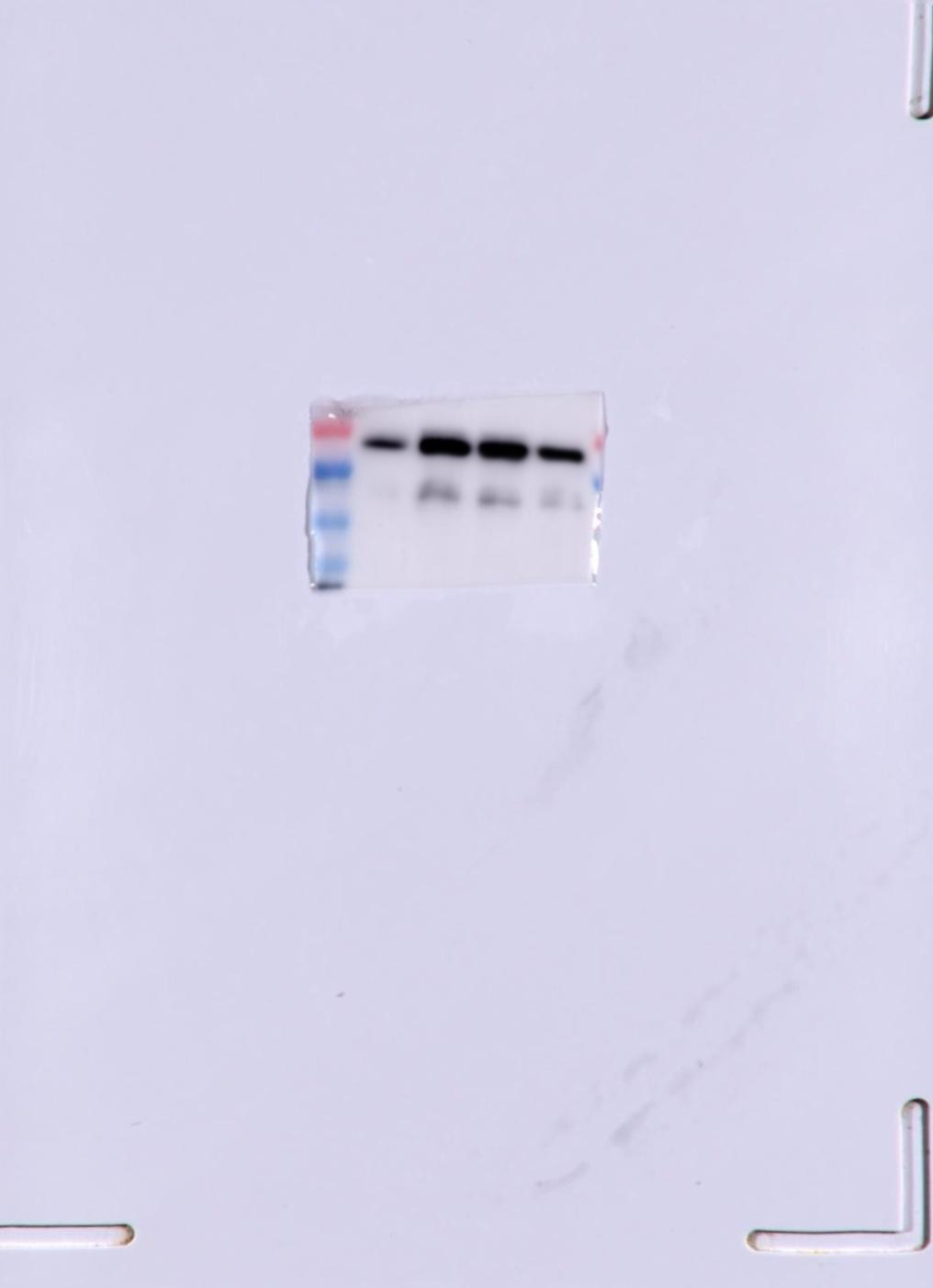


p-NF-κB-p65

Besides marker in the picture, from left to right are the Sham, AAA, AAA+DMSO, and AAA+inhibitor groups in sequence.


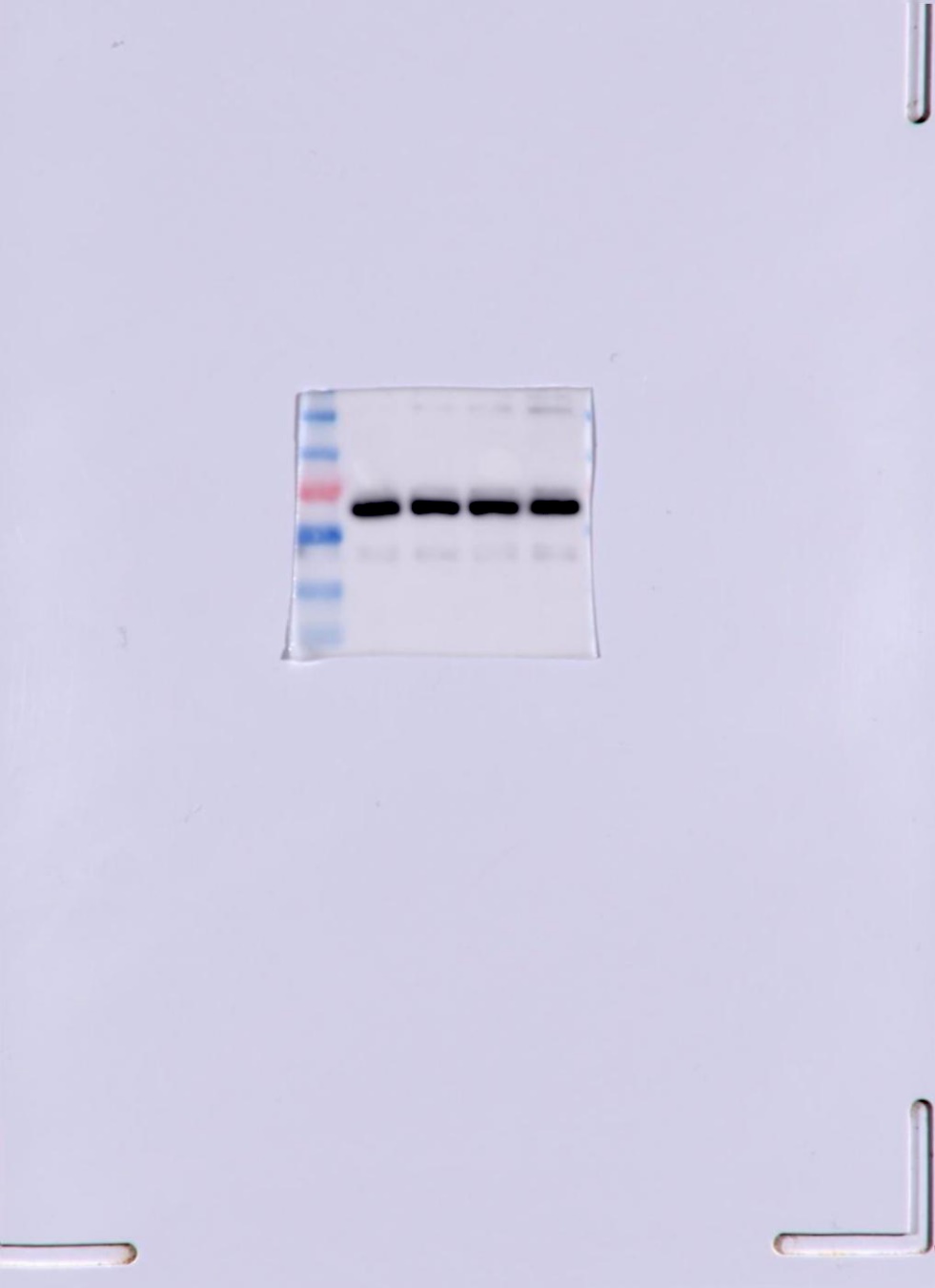


NF-κB-p65

Besides marker in the picture, from left to right are the Sham, AAA, AAA+DMSO, and AAA+inhibitor groups in sequence.


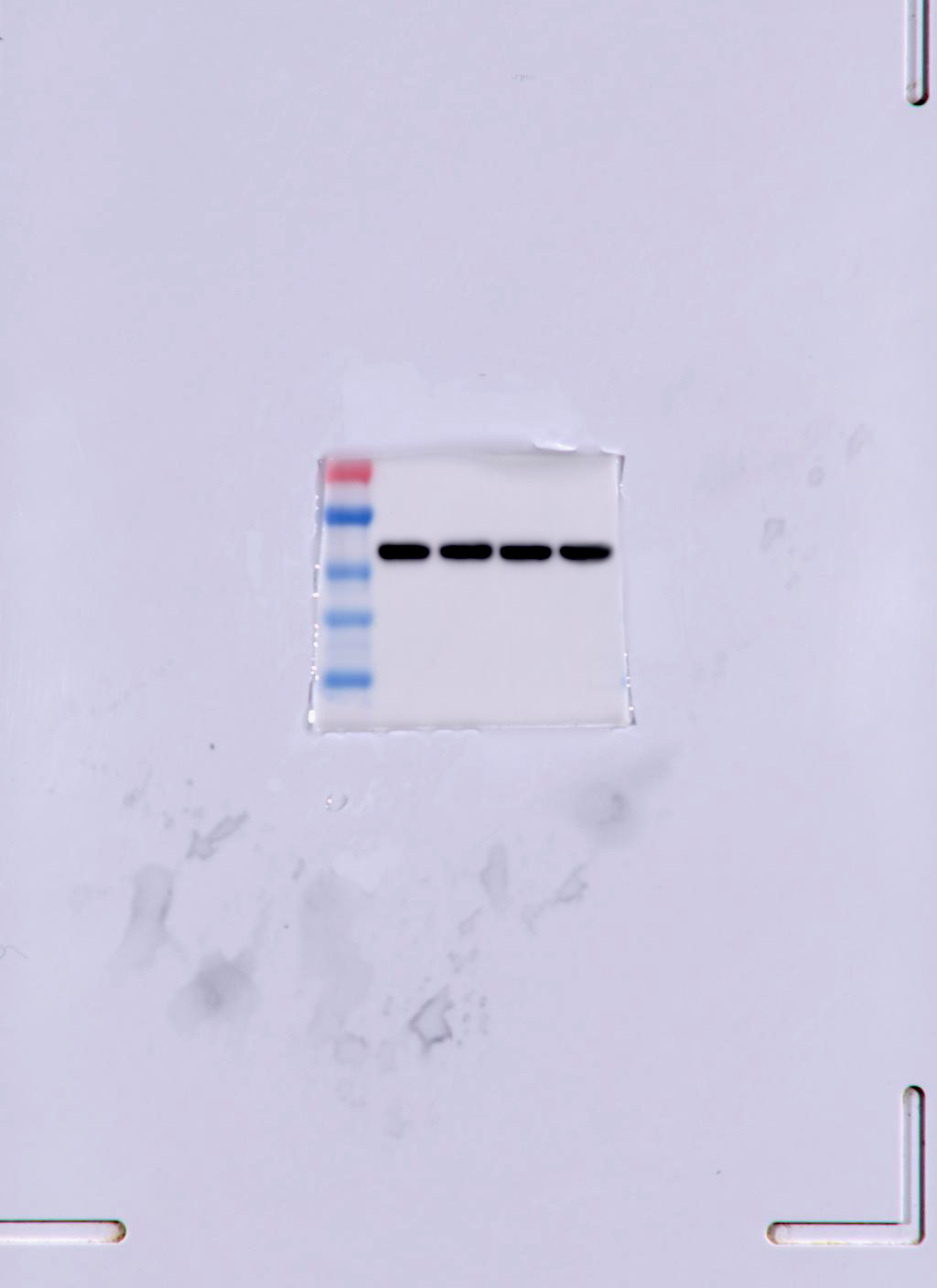


β-actin

Besides marker in the picture, from left to right are the Sham, AAA, AAA+DMSO, and AAA+inhibitor groups in sequence.

Figure 4H


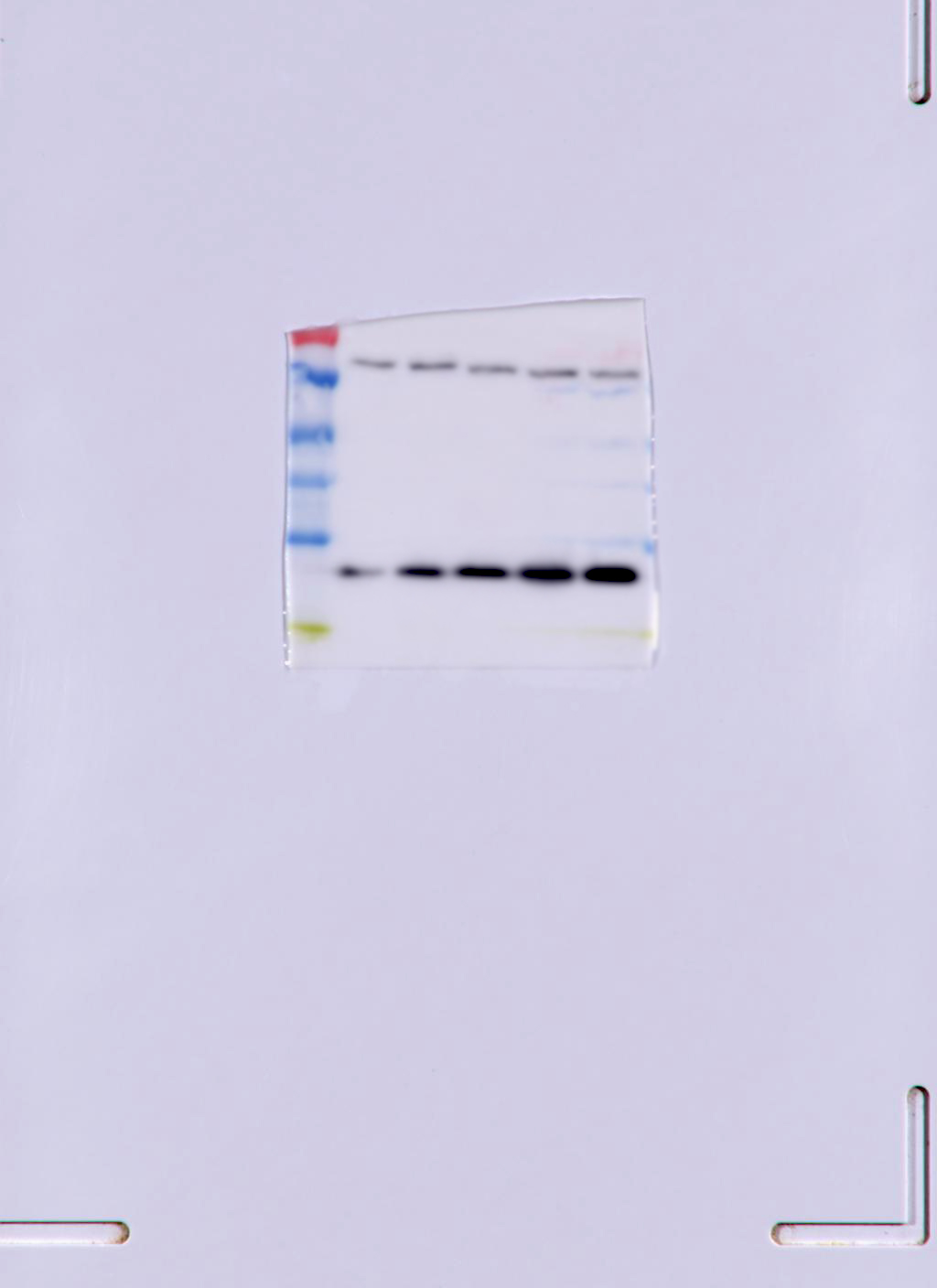


ASC

Besides marker in the picture, from left to right are the Control, 6h, 12h, 24h and 48h groups in sequence.


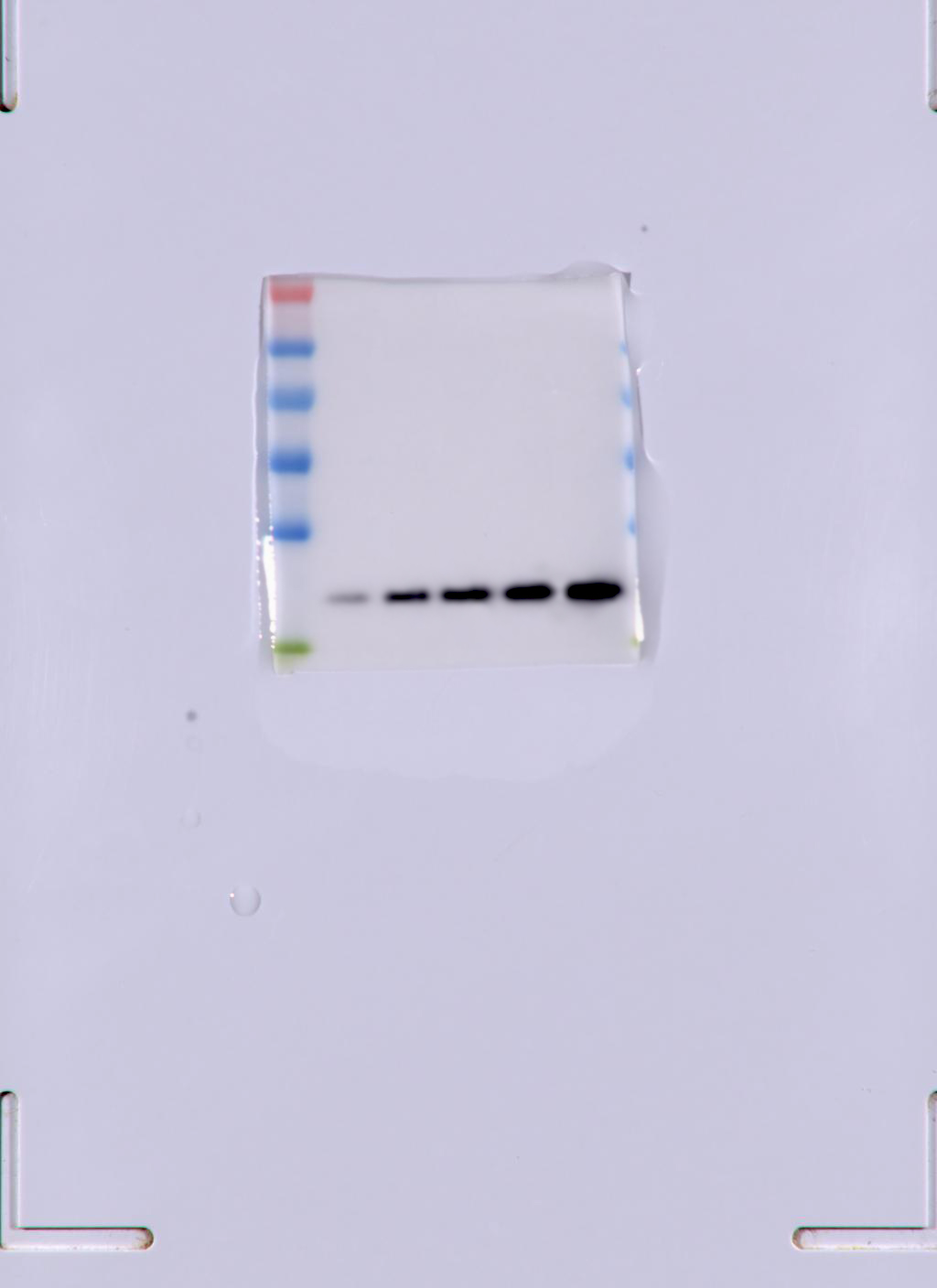


caspase-1

Besides marker in the picture, from left to right are the Control, 6h, 12h, 24h and 48h groups in sequence.


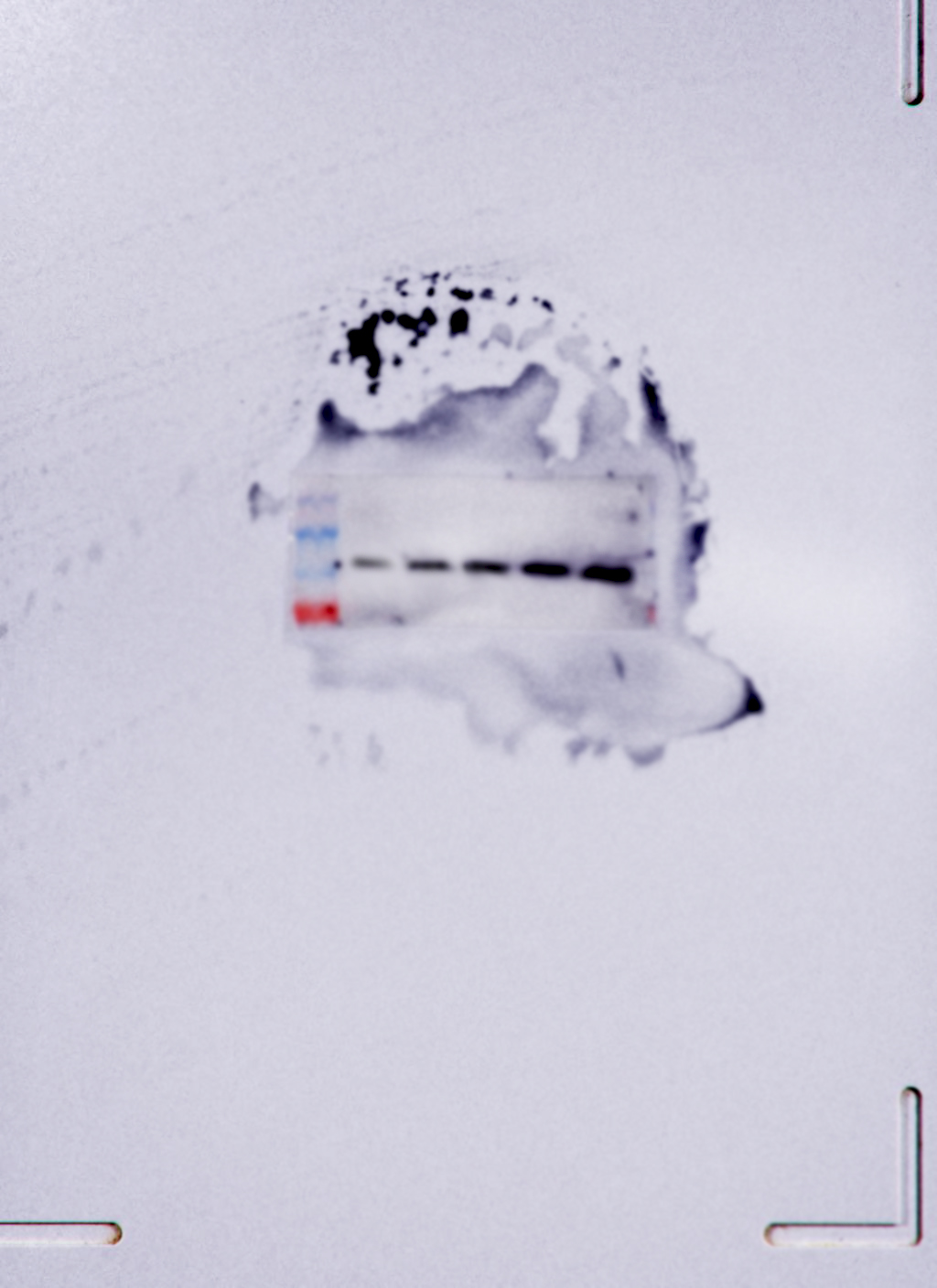


NLRP3

Besides marker in the picture, from left to right are the Control, 6h, 12h, 24h and 48h groups in sequence.


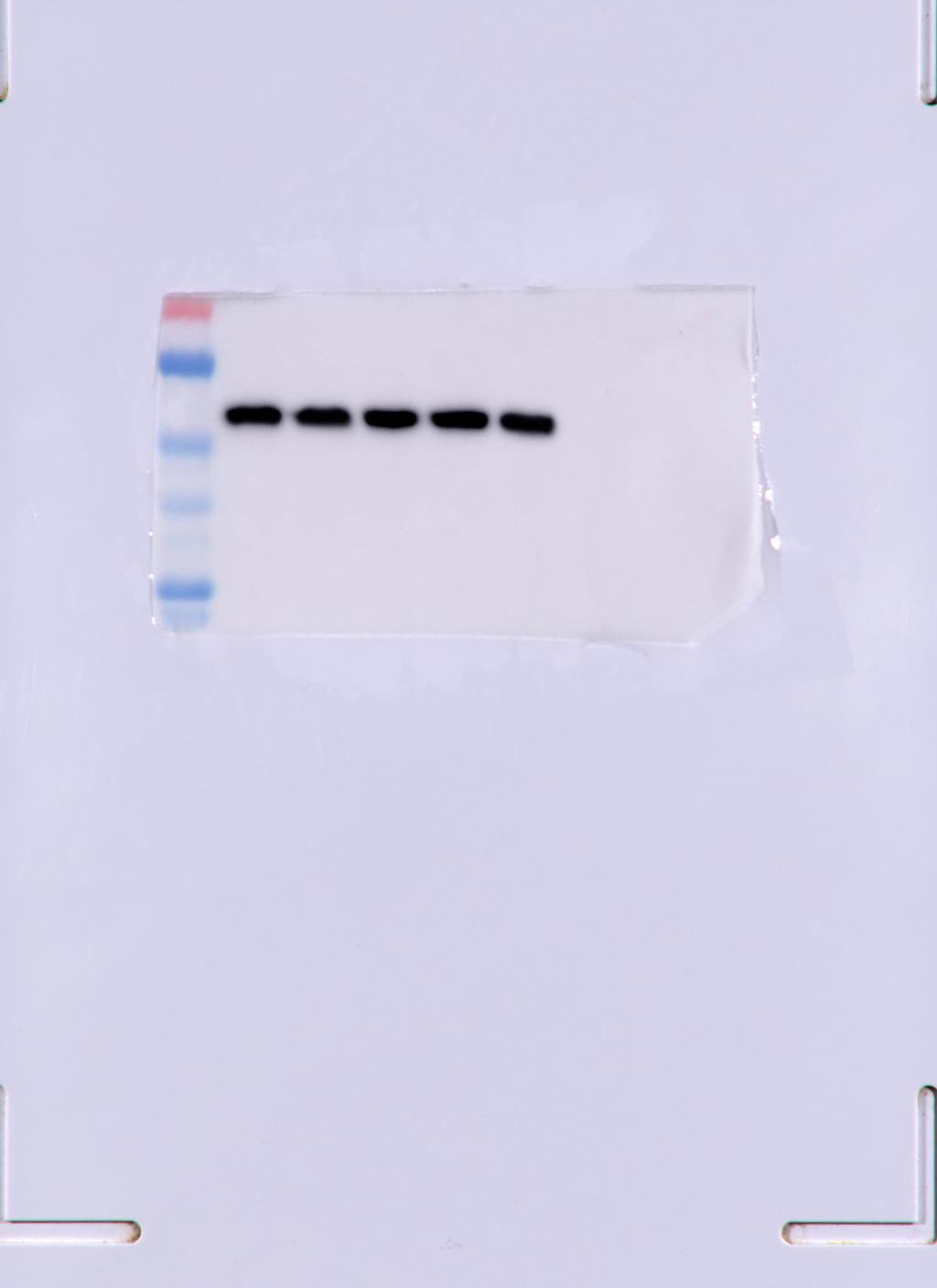


β-actin

Besides marker in the picture, from left to right are the Control, 6h, 12h, 24h and 48h groups in sequence.

Figure 5C


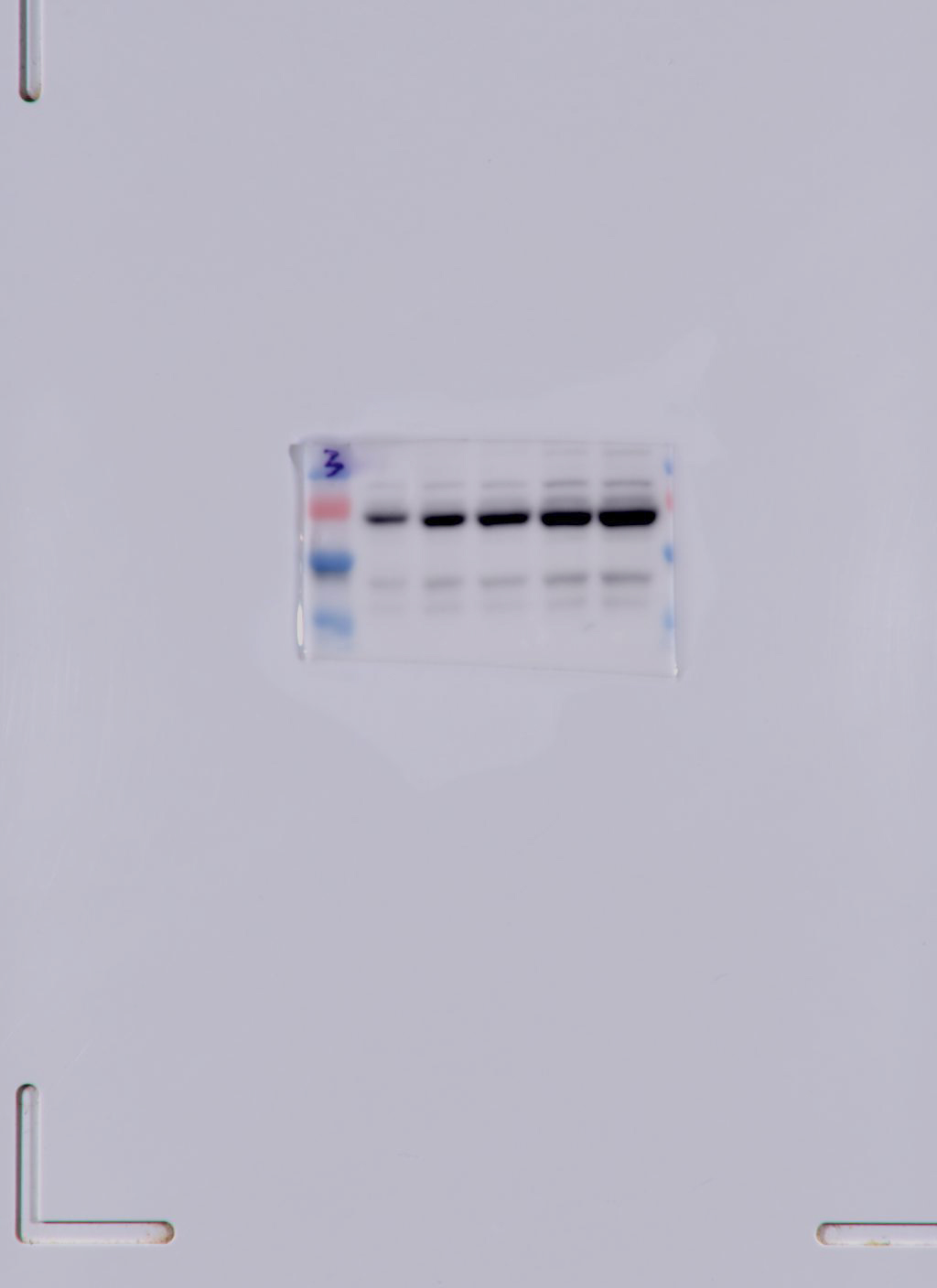

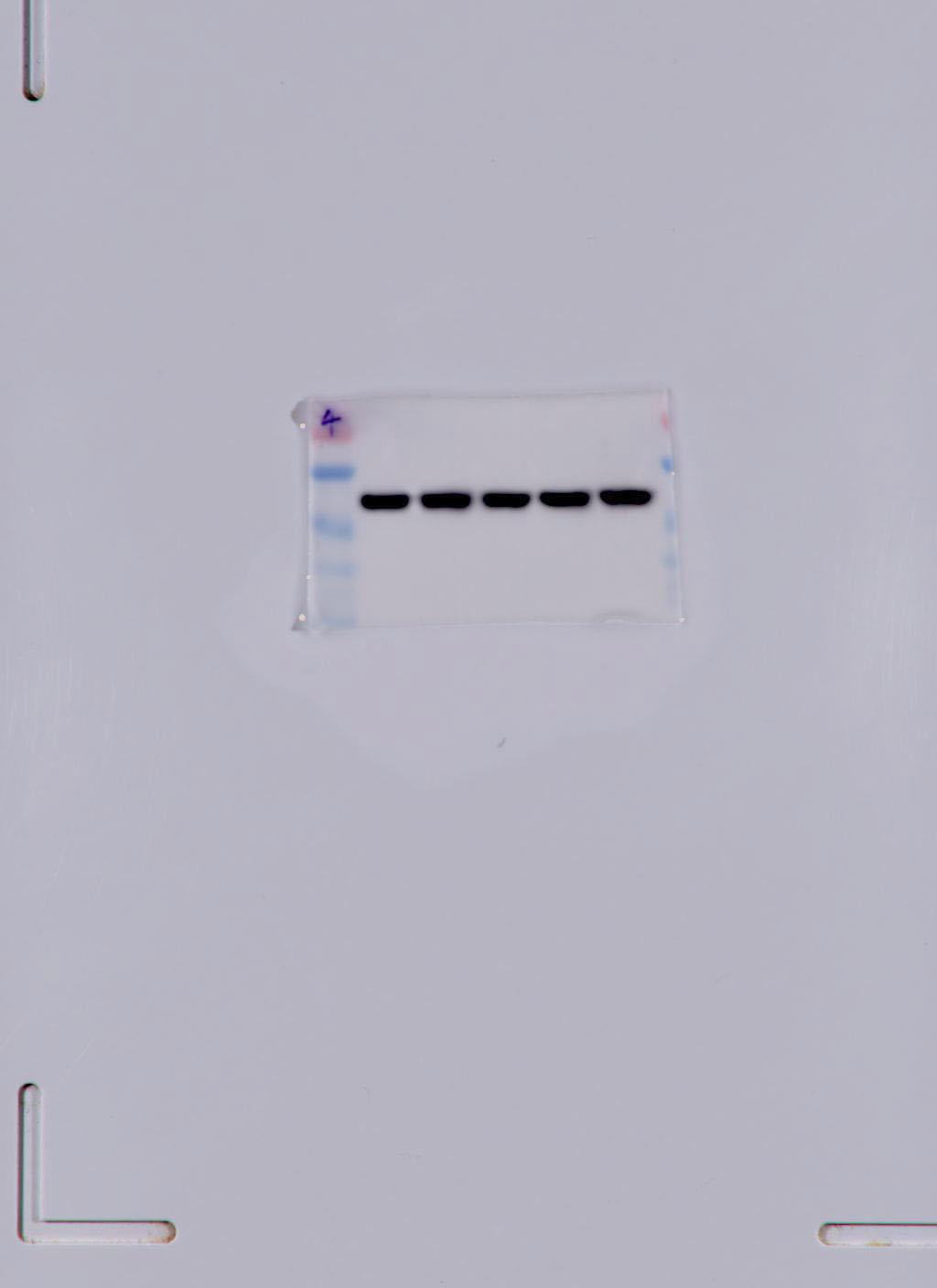


ALOX5

Besides marker in the picture, from left to right are the Control, 6h, 12h, 24h and 48h groups in sequence.

β-actin

Besides marker in the picture, from left to right are the Control, 6h, 12h, 24h and 48h groups in sequence.

Figure 7F


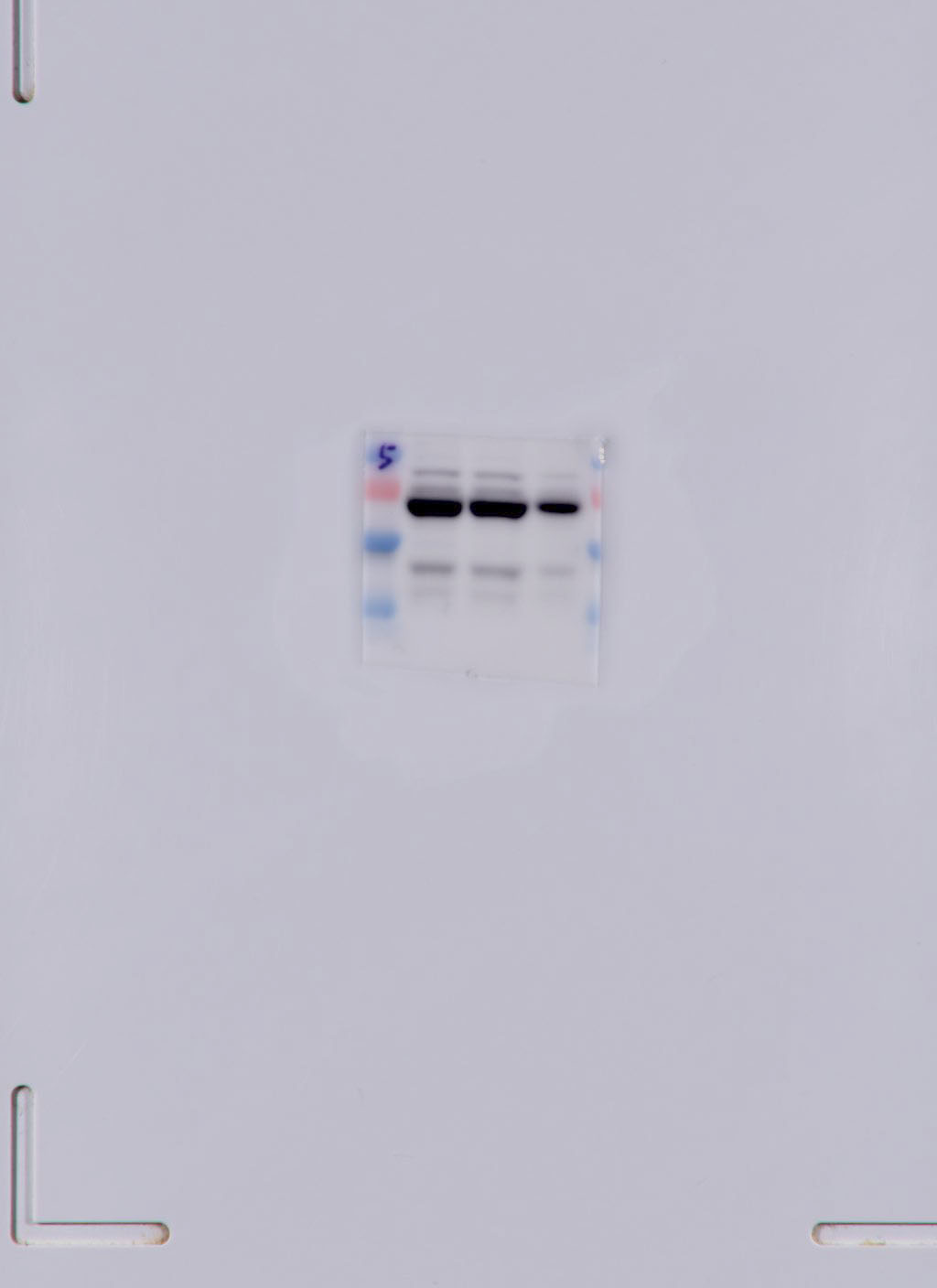

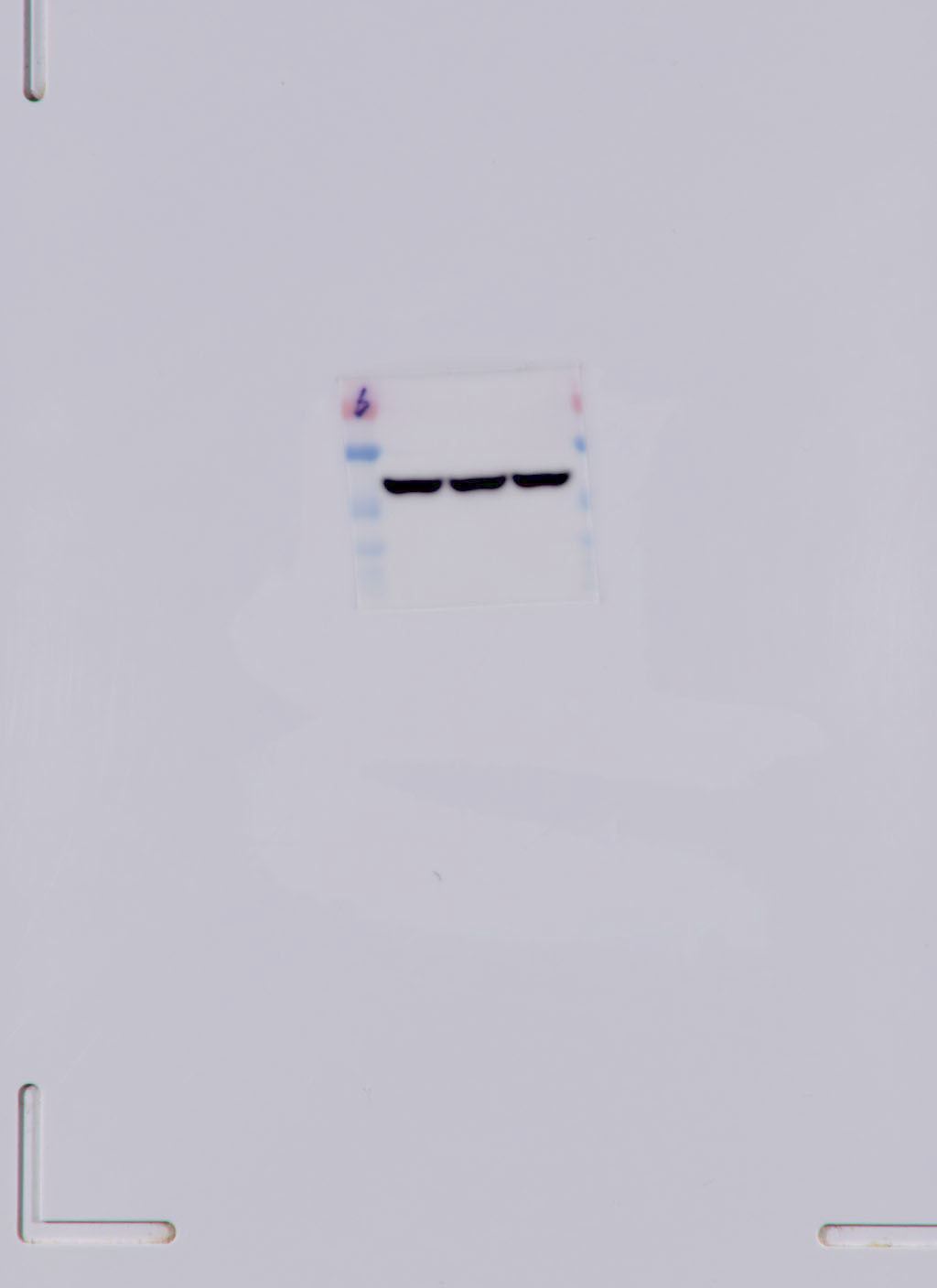


ALOX5

Besides marker in the picture, from left to right are the Control, si-NC, and si-ALOX5 groups in sequence.

β-actin

Besides marker in the picture, from left to right are the Control, si-NC, and si-ALOX5 groups in sequence.

Figure 8B


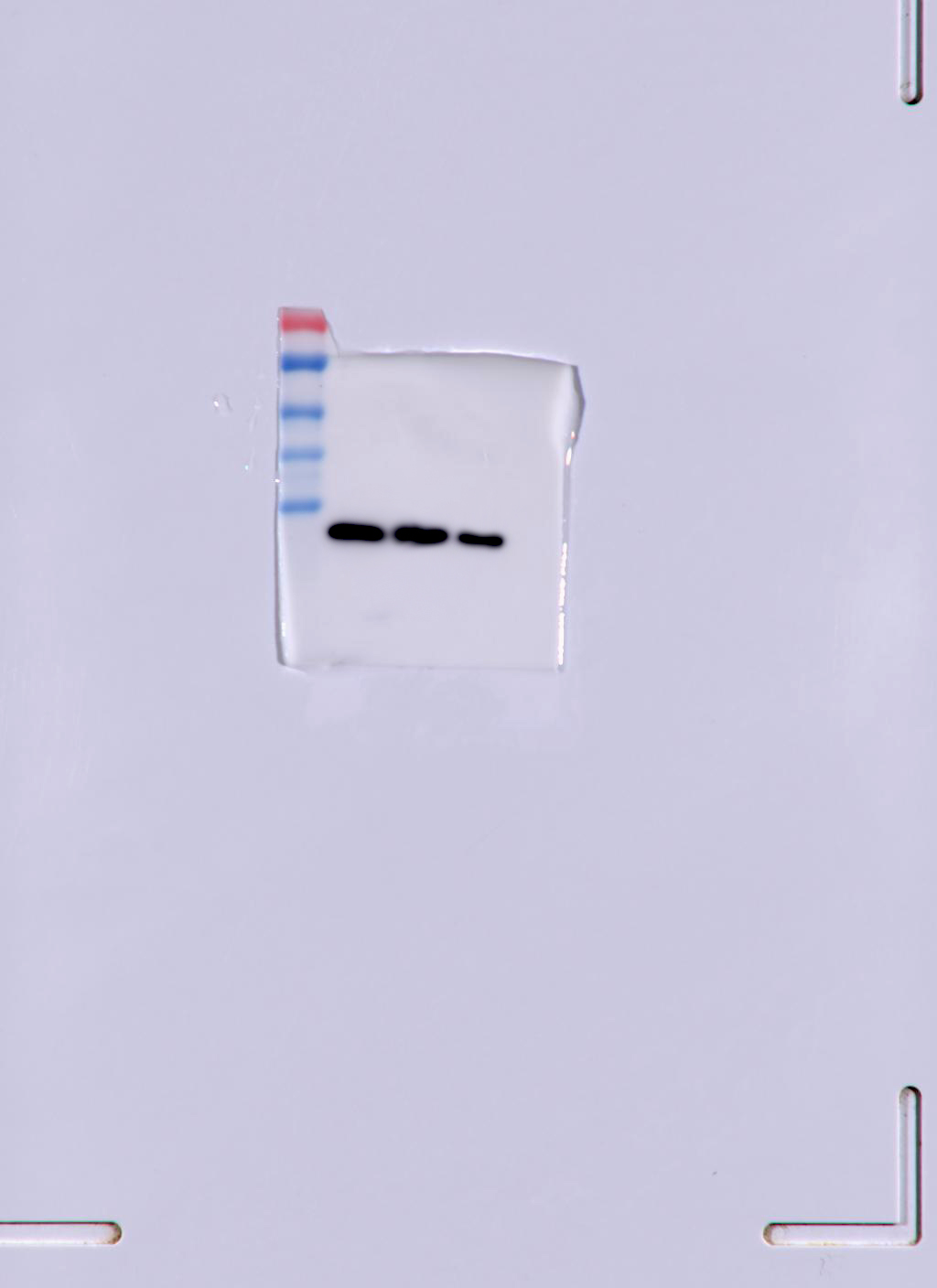


ASC

Besides marker in the picture, from left to right are the Ang Ⅱ, Ang Ⅱ+si-NC, and Ang Ⅱ+si-ALOX5 groups in sequence.


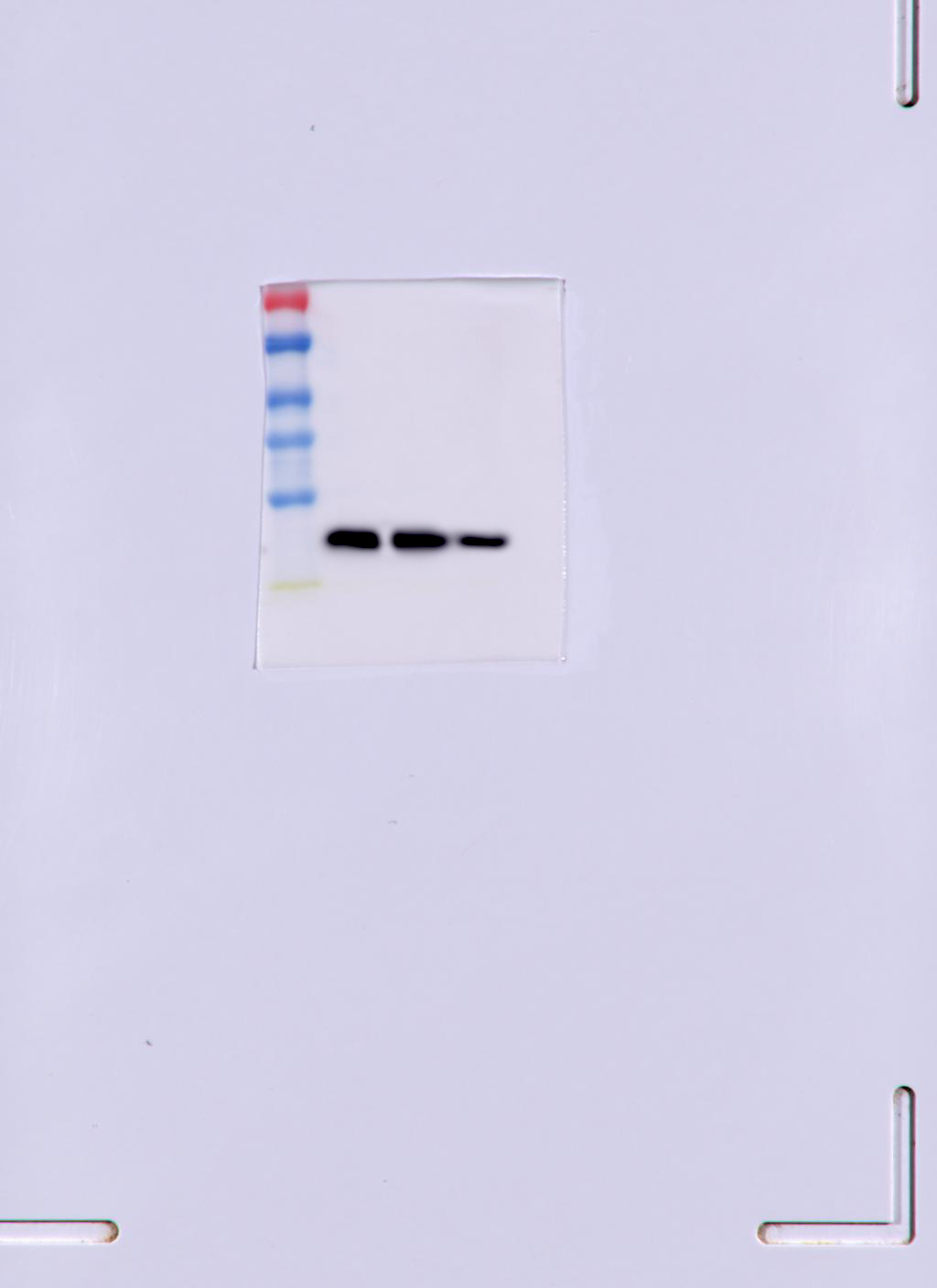


caspase-1

Besides marker in the picture, from left to right are the Ang Ⅱ, Ang Ⅱ+si-NC, and Ang Ⅱ+si-ALOX5 groups in sequence.


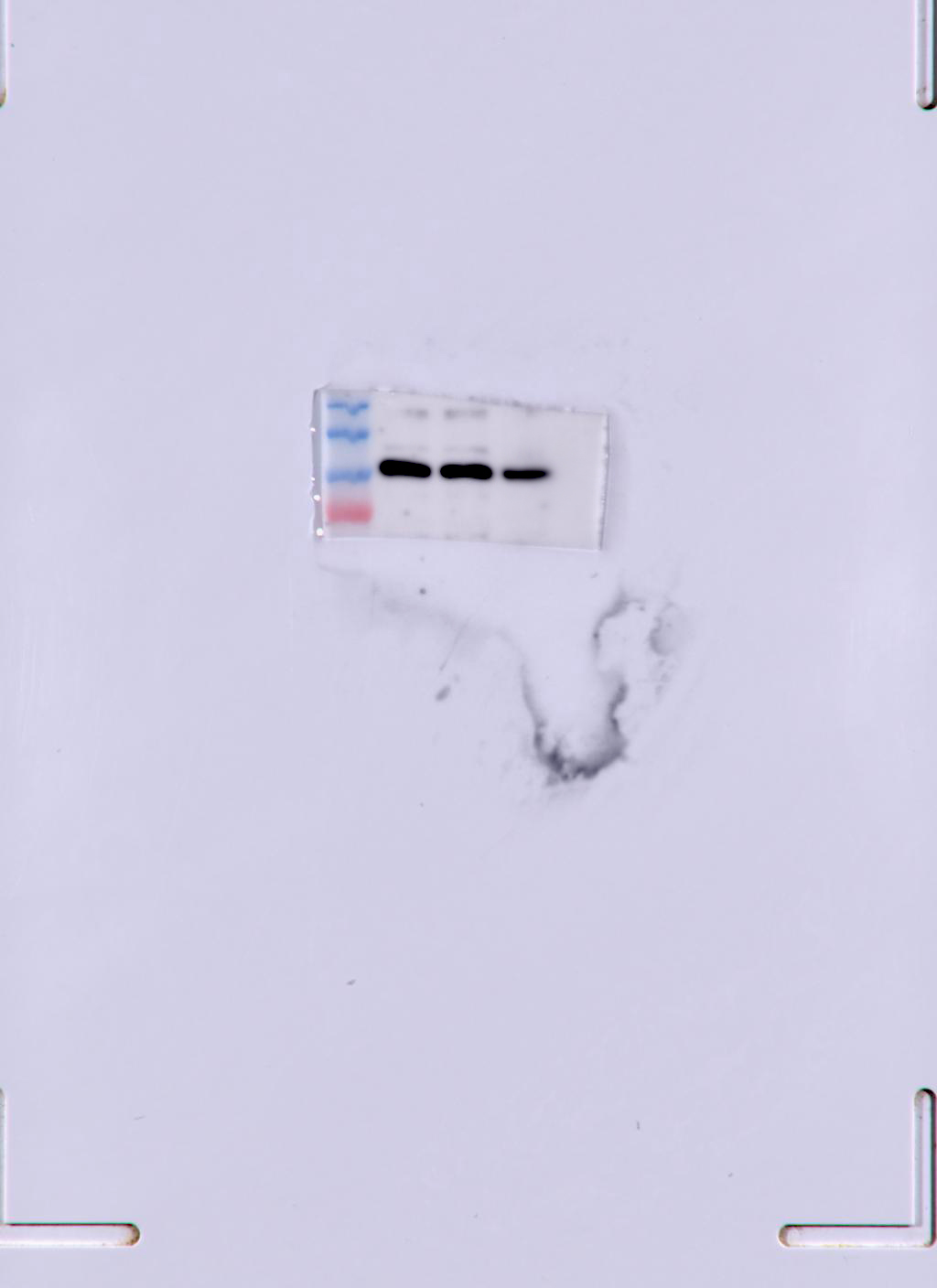


NLRP3

Besides marker in the picture, from left to right are the Ang Ⅱ, Ang Ⅱ+si-NC, and Ang Ⅱ+si-ALOX5 groups in sequence.


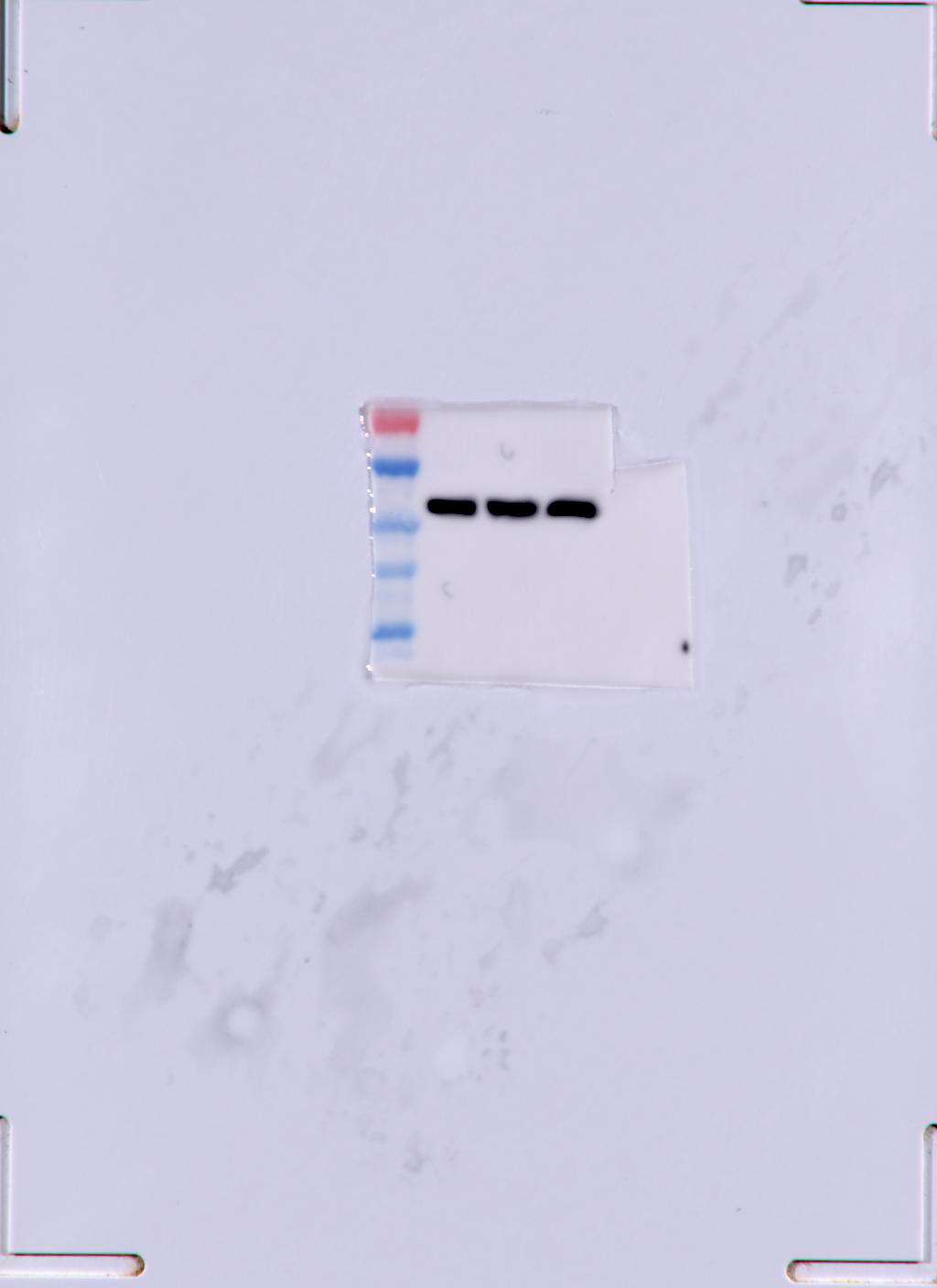


β-actin

Besides marker in the picture, from left to right are the Ang Ⅱ, Ang Ⅱ+si-NC, and Ang Ⅱ+si-ALOX5 groups in sequence.

Figure 8F


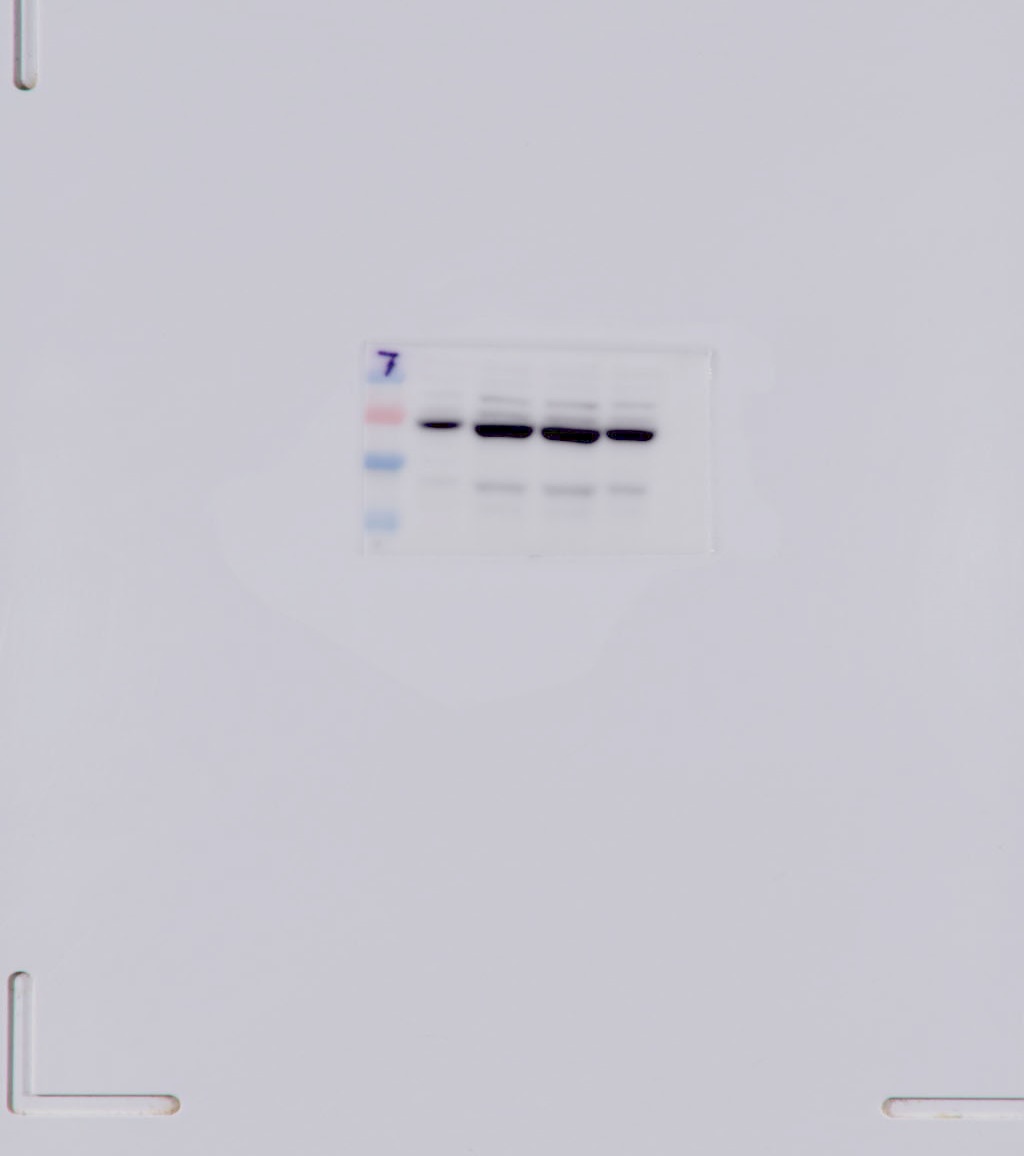

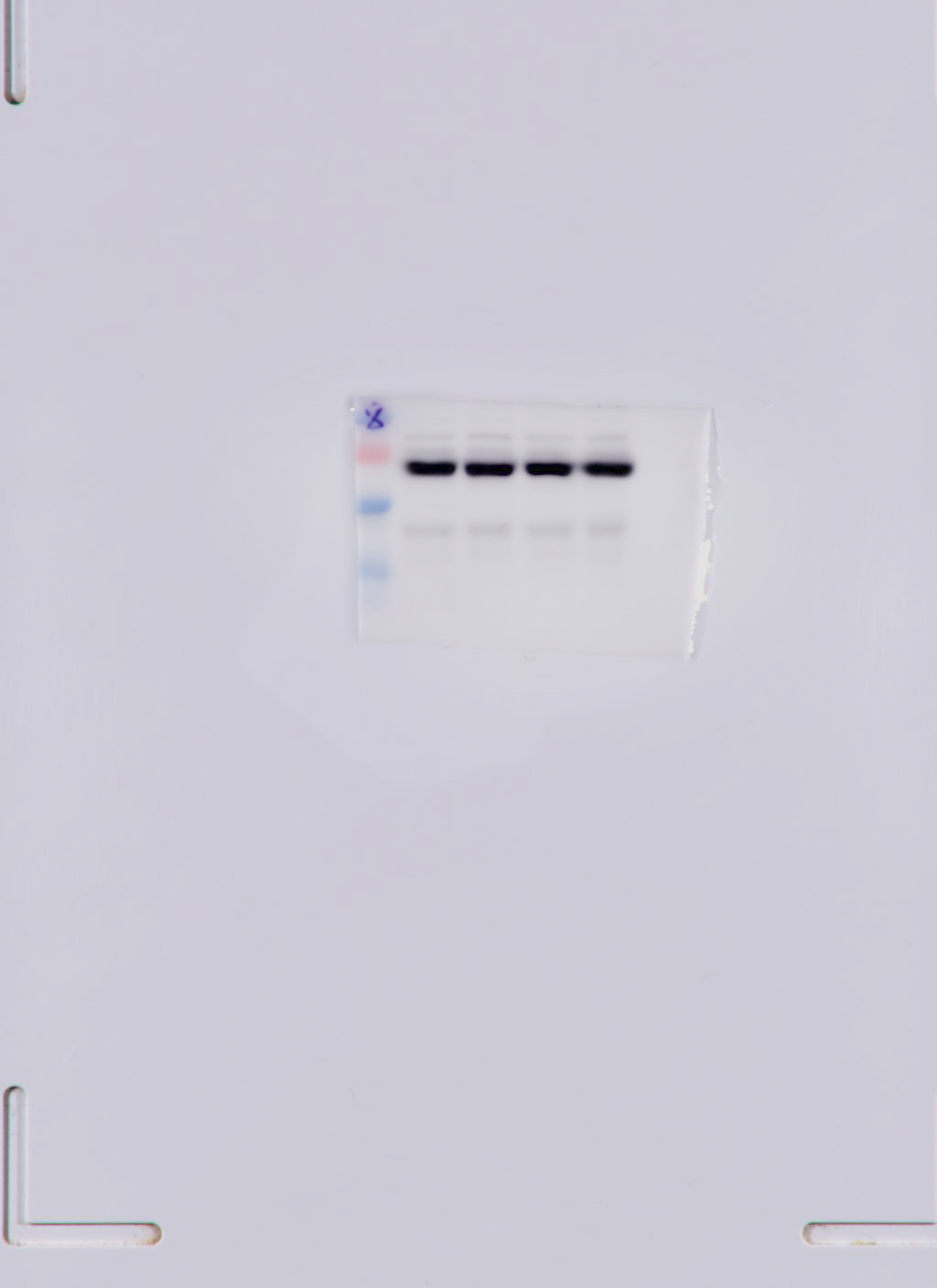

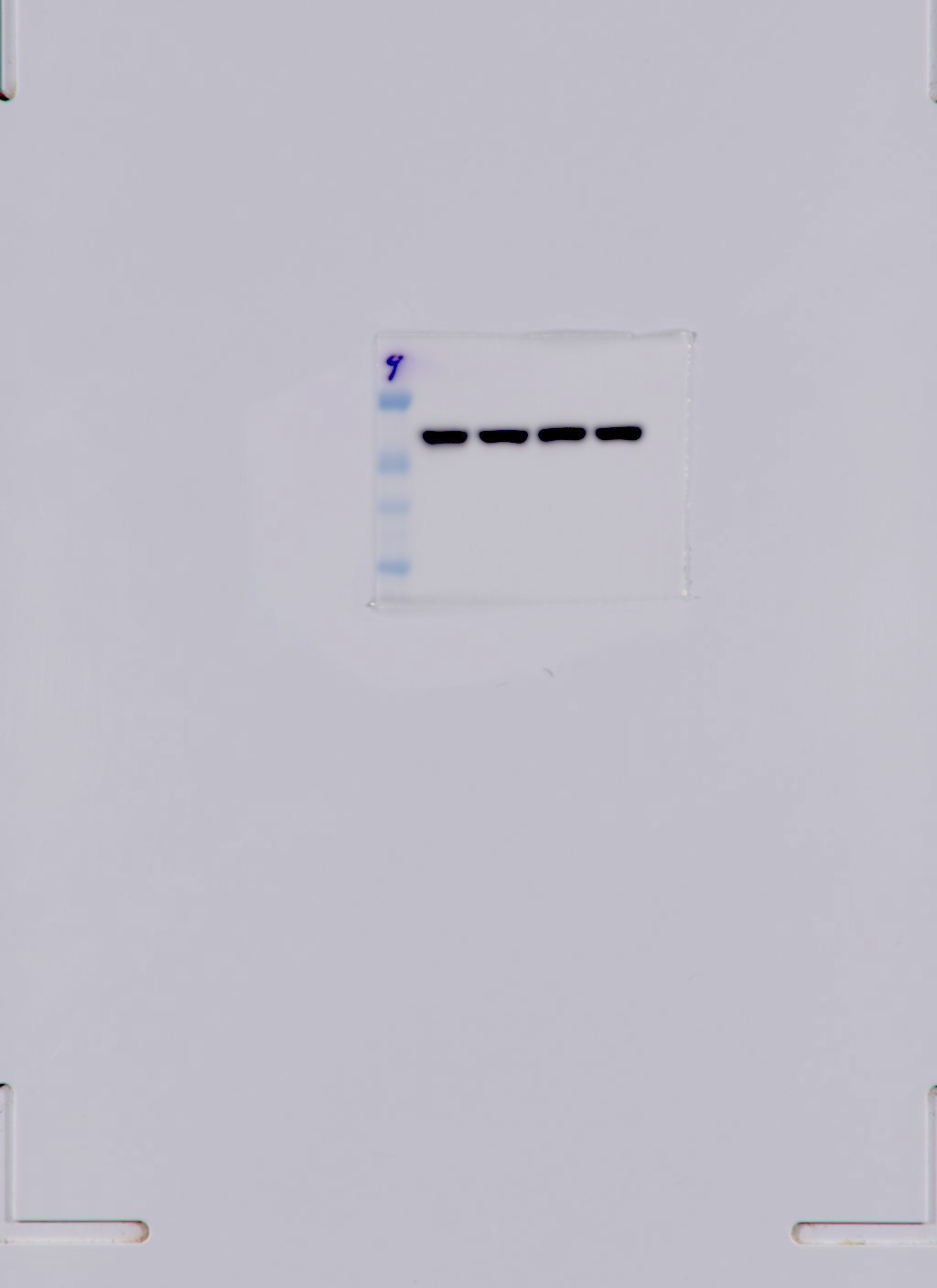


p-NF-κB-p65

Besides marker in the picture, from left to right are the Control, Ang Ⅱ, Ang Ⅱ+si-NC, and Ang Ⅱ+si-ALOX5 groups in sequence.

NF-κB-p65

Besides marker in the picture, from left to right are the Control, Ang Ⅱ, Ang Ⅱ+si-NC, and Ang Ⅱ+si-ALOX5 groups in sequence.

β-actin

Besides marker in the picture, from left to right are the Control, Ang Ⅱ, Ang Ⅱ+si-NC, and Ang Ⅱ+si-ALOX5 groups in sequence.

Figure 10A


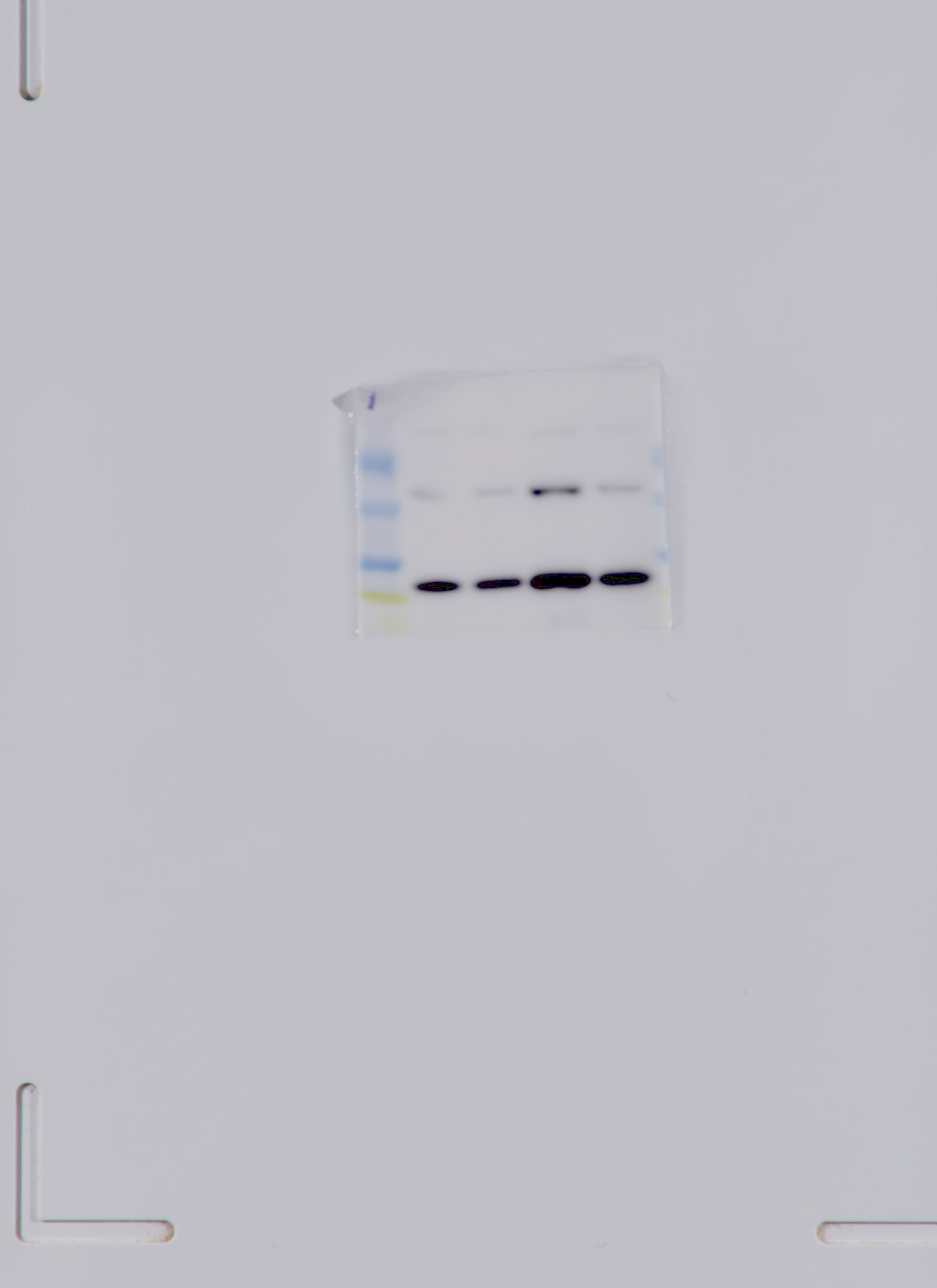

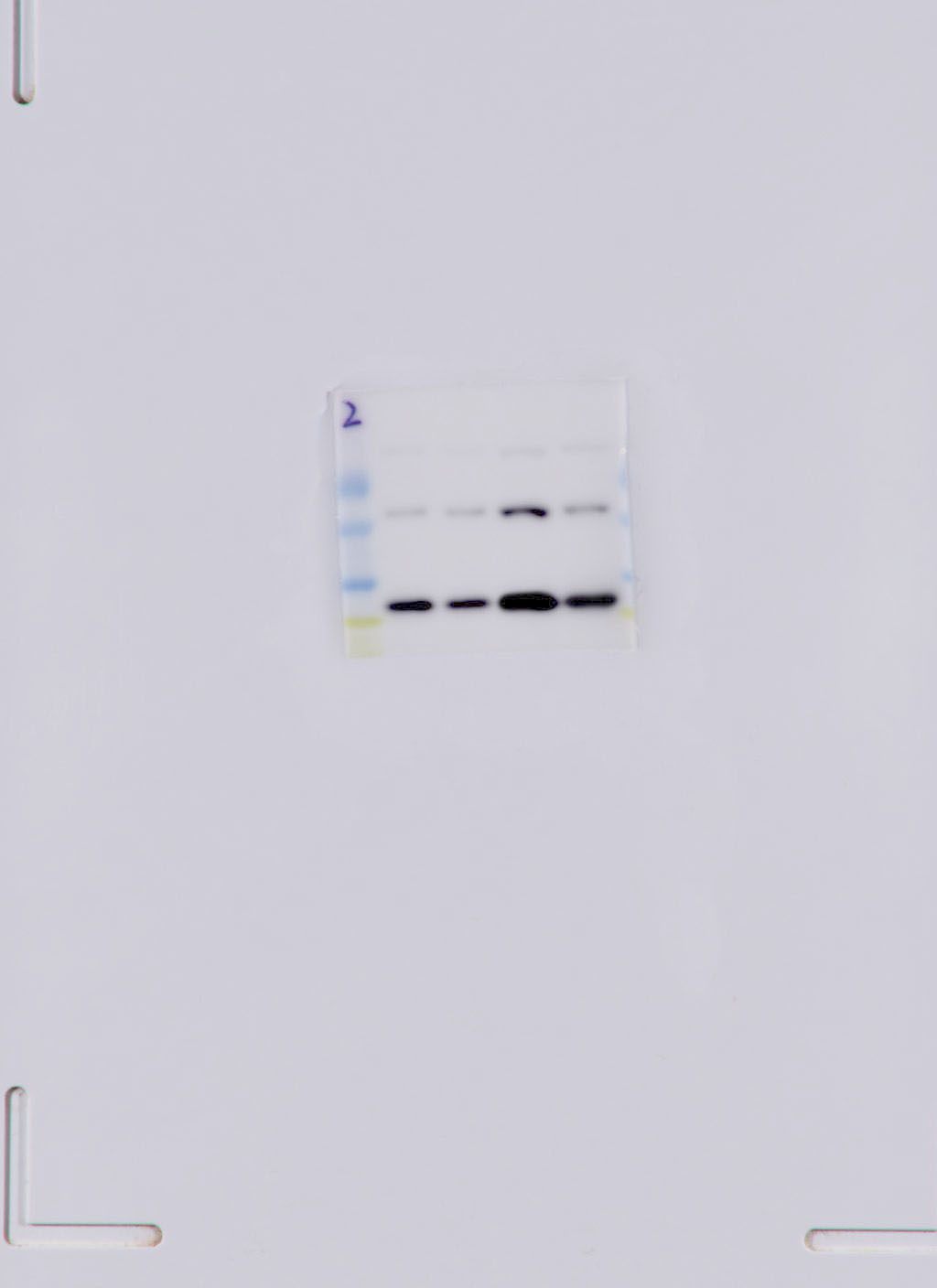

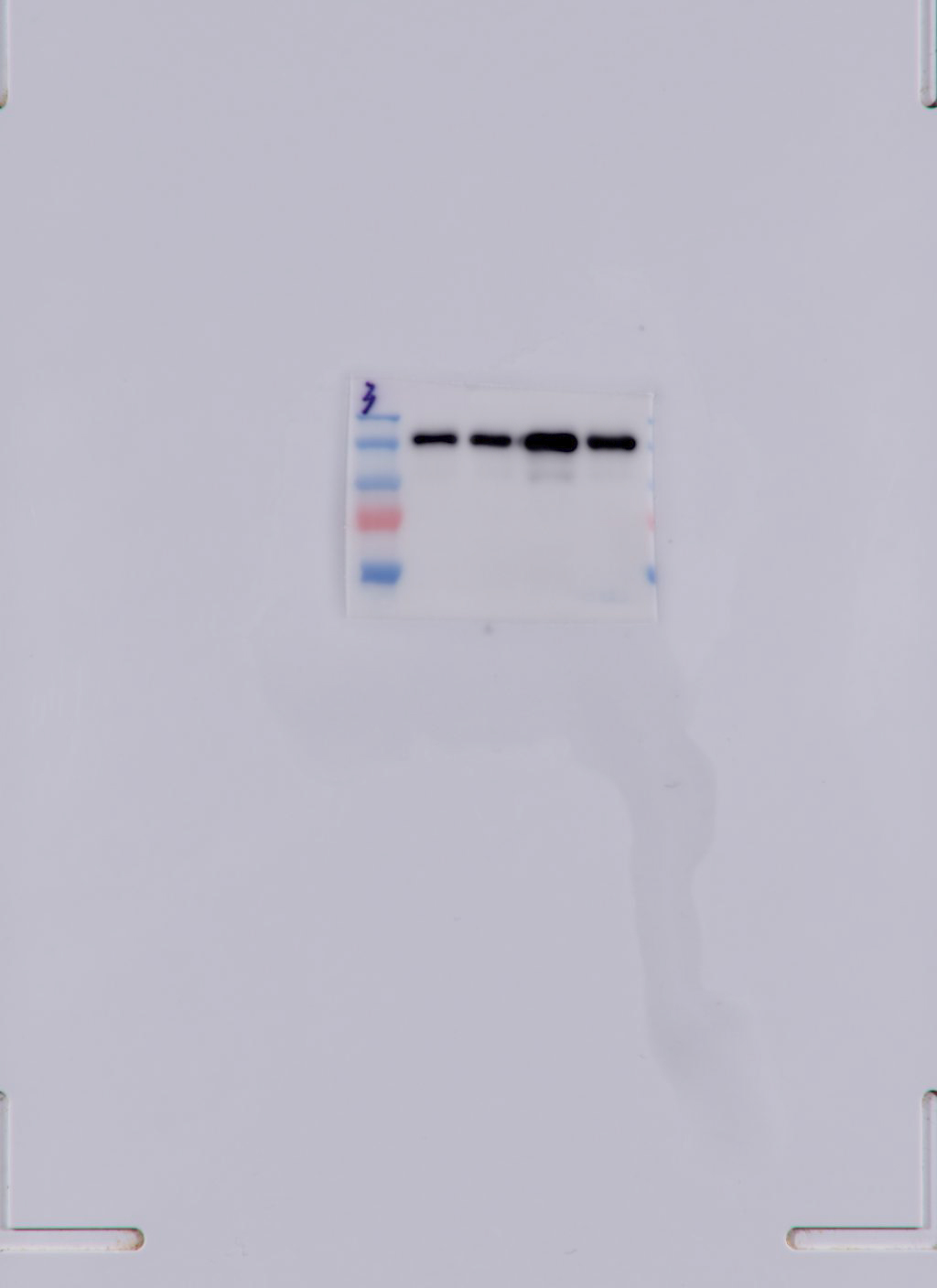


ASC

Besides marker in the picture, from left to right are the Ang Ⅱ, Ang Ⅱ+pc-NC, Ang Ⅱ+pc-ALOX5, and Ang Ⅱ+pc-ALOX5+BAY groups in sequence.

caspase-1

Besides marker in the picture, from left to right are the Ang Ⅱ, Ang Ⅱ+pc-NC, Ang Ⅱ+pc-ALOX5, and Ang Ⅱ+pc-ALOX5+BAY groups in sequence.

NLRP3

Besides marker in the picture, from left to right are the Ang Ⅱ, Ang Ⅱ+pc-NC, Ang Ⅱ+pc-ALOX5, and Ang Ⅱ+pc-ALOX5+BAY groups in sequence.


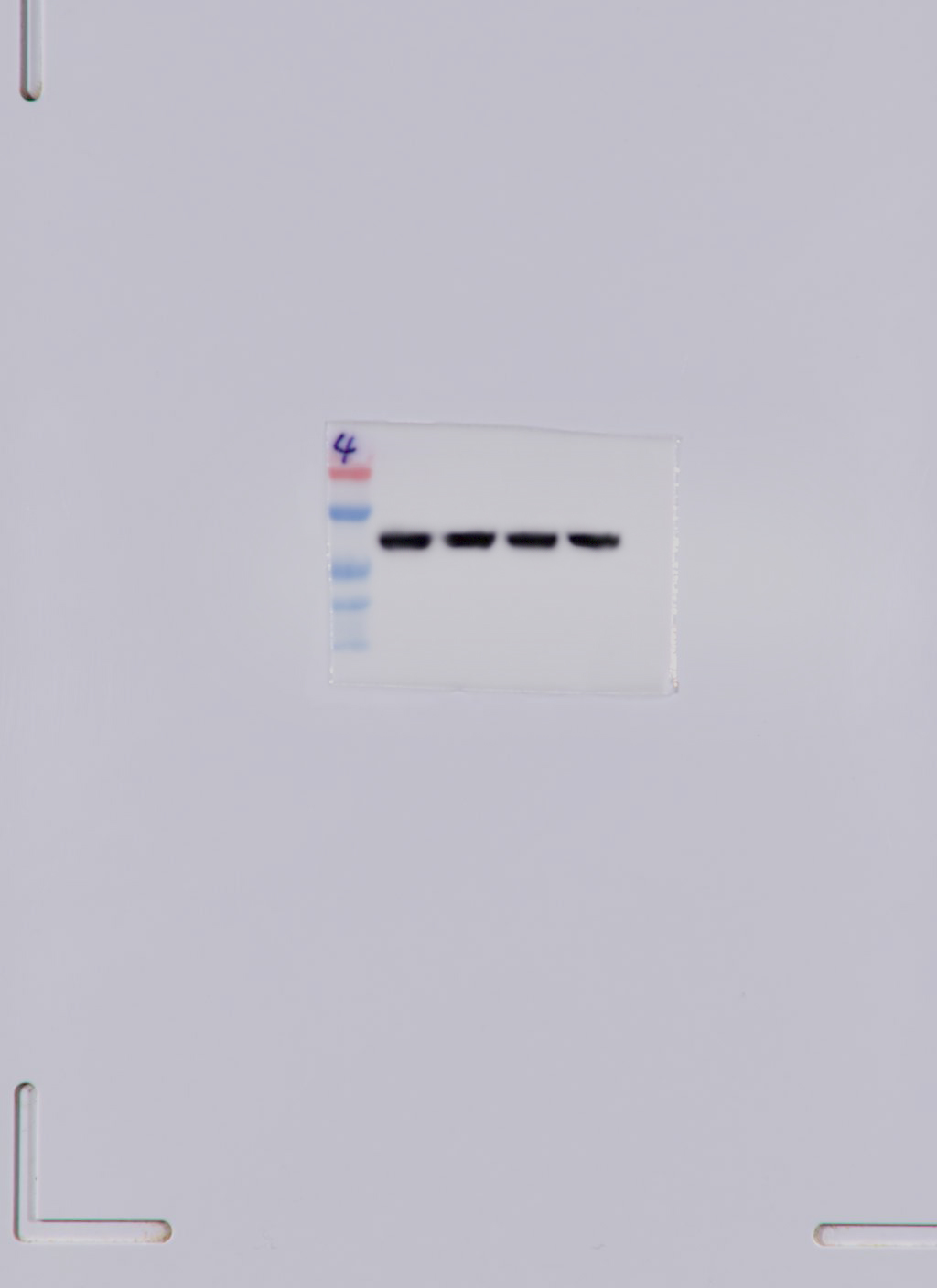


β-actin

Besides marker in the picture, from left to right are the Ang Ⅱ, Ang Ⅱ+pc-NC, Ang Ⅱ+pc-ALOX5, and Ang Ⅱ+pc-ALOX5+BAY groups in sequence.

Figure 11A
